# Advanced Data Analysis for Fluorescence-Lifetime Single-Molecule Localization Microscopy

**DOI:** 10.3389/fbinf.2021.740281

**Published:** 2021-11-19

**Authors:** Jan Christoph Thiele, Oleksii Nevskyi, Dominic A. Helmerich, Markus Sauer, Jörg Enderlein

**Affiliations:** ^1^ Third Institute of Physics—Biophysics, Georg August University, Göttingen, Germany; ^2^ Department of Biotechnology and Biophysics, Biocenter, Julius-Maximilians-Universität Würzburg, Würzburg, Germany; ^3^ Cluster of Excellence “Multiscale Bioimaging: from Molecular Machines to Networks of Excitable Cells” (MBExC), Georg August University, Göttingen, Germany

**Keywords:** FLIM (fluorescence lifetime imaging microscopy), CRLB (Cramér-Rao lower bound) analysis, fluoresence lifetime fitting, super-resolution microscopy, lifetime uncertainty, SMLM (single molecule localisation microscopy)

## Abstract

Fluorescence-lifetime single molecule localization microscopy (FL-SMLM) adds the lifetime dimension to the spatial super-resolution provided by SMLM. Independent of intensity and spectrum, this lifetime information can be used, for example, to quantify the energy transfer efficiency in Förster Resonance Energy Transfer (FRET) imaging, to probe the local environment with dyes that change their lifetime in an environment-sensitive manner, or to achieve image multiplexing by using dyes with different lifetimes. We present a thorough theoretical analysis of fluorescence-lifetime determination in the context of FL-SMLM and compare different lifetime-fitting approaches. In particular, we investigate the impact of background and noise, and give clear guidelines for procedures that are optimized for FL-SMLM. We do also present and discuss our public-domain software package “Fluorescence-Lifetime TrackNTrace,” which converts recorded fluorescence microscopy movies into super-resolved FL-SMLM images.

## 1 Introduction

The advent of super-resolution microscopy (Hell, 2007; Huang et al., 2009) has revolutionized optical microscopy over the last ca. 30 years, pushing the limits of spatial resolution by three orders of magnitude down to the molecular length scale. The first of these super-resolution methods was STimulated Emission Depletion (STED) microscopy (Hell and Wichmann, 1994; Klar et al., 2000), developed by Stefan Hell and co-workers since the nineties of the last century, and later extended to Ground State Depletion IMaging (GSDIM) (Fölling et al., 2008; Hell, 2009) and REversible Saturable OpticaL Fluorescence Transitions (RESOLFT) imaging (Keller et al., 2007; Schwentker et al., 2007). This spurred also the development of alternative methods that use single-molecule localization in wide-field images (Single-Molecule Localization Microscopy or SMLM) (Klein et al., 2014). Among these methods are PhotoActivated Localization Microscopy (PALM) (Betzig et al., 2006), Stochastic Optical Reconstruction Microscopy (STORM) (Rust et al., 2006), fluorescence PALM (fPALM) (Hess et al., 2006), direct STORM (dSTORM) (Van de Linde et al., 2011), Point Accumulation for Imaging in Nanoscale Topography (PAINT) microscopy (Sharonov and Hochstrasser, 2006), and its most common variant DNA-PAINT (Schnitzbauer et al., 2017; Auer et al., 2018). These methods rely on the fact that one can localize the center position of an emitting molecule with much higher accuracy than the width of the molecule’s image, the latter being defined by the optical resolution of the used microscope. Roughly speaking, this localization accuracy scales as the diffraction-limited resolution divided by the square root of the number of detected photons, so that, for example, a molecule that delivers 10^4^ detectable photons can be localized ca. 100 times better than the classical resolution limit (neglecting here, for simplicity, all kinds of details such as noise, background, or detector pixelation) (Ram et al., 2006). By recording many images of well-separated molecules (by using fluorescent labels that can be switched between non-fluorescent and fluorescent states), one can generate a super-resolved image, the resolution of which is only limited by the number of photons detectable from a single molecule.

One powerful extension of fluorescence microscopy is fluorescence lifetime imaging microscopy (FLIM) (Bastiaens and Squire, 1999; van Munster and Gadella, 2005; Chang et al., 2007) which measures, besides the intensity of the fluorescence signal, also its lifetime. This lifetime information can be, for example, used for multiplexing by using fluorophores with different lifetimes (Niehörster et al., 2016), for Förster Resonance Energy Transfer (FRET) imaging (Llères et al., 2017), or to probe different environmental characteristics when using fluorophores that change their lifetime as function of specific parameters (e.g. pH, ion concentration, viscosity) (Klymchenko, 2017). The two most common FLIM techniques are based on a confocal microscope equipped with a pulsed laser source, single-photon sensitive detectors and electronics for Time-Correlated Single Photon Counting (TCSPC) (Becker, 2005; O’Connor, 2012), or on phase fluorometry using a time-modulated excitation source and a wide-field detector with time-modulated detection gain (Venetta, 1959; Spencer and Weber, 1969; Dong et al., 1995). However, both these approaches are usually not suitable for SMLM: Confocal microscopy was until recently rarely used for SMLM due to the limited frame rate, and phase-fluorometry systems are by far to insensitive for single-molecule imaging. In contrast, single-molecule sensitive wide-field detectors such as emCCD or sCMOS cameras that are generally used for SMLM do not provide any lifetime information. Only recently, it has been shown that one can use rapid-scanning confocal TCSPC microscopy for fluorescence-lifetime SMLM (FL-SMLM) (Thiele et al., 2020). In this case, one rapidly records confocal images with single-molecule sensitivity and then analyses the stack of recorded scan images in the same way as is done in conventional wide-field SMLM. A drawback is that the light-throughput (or dwell-time per position) in a confocal microscopy is much lower than that of a camera-equipped wide-field microscope, but the advantage is that one can obtain the lifetime information for each imaged and registered molecule, and that the z-sectioning capability of the confocal microscope can help to do SMLM even deeper into a sample, where out-of-focus background light becomes a problem. Alternatively to confocal TCSPC microscopy, new single-photon sensitive wide-field cameras that can measure lifetime information with TCSPC are more and more emerging. One type of such cameras is based on an array of single-photon avalanche diodes (Ulku et al., 2018; Morimoto et al., 2020) and shows great promise for future SMLM applications. A second type of wide-field TCSPC detectors is the commercially available LINCam (PhotonScore GmbH, Magdeburg, Germany), that has been successfully used for FL-SMLM (Oleksiievets et al., 2020). Although this system has a relatively low quantum yield of detection (5–15%), it shows nearly complete absence of any readout or other camera noise, thus assuring sufficient high signal-to-background ratios for successful single-molecule imaging.

Thus, with the advent of FL-SMLM, the question arises what is the most optimal and efficient way of TCSPC-based fluorescence-lifetime determination for SMLM. Within the context of single-molecule spectroscopy, different fitting methods have been discussed and evaluated with experimental data, indicating that maximum likelihood estimations outperform least-square minimization techniques (Maus et al., 2001; Santra et al., 2016), and theoretical limits have been derived analytically (for background-free case) (Köllner and Wolfrum, 1992) and numerically (for a large range of experimental parameters) (Bouchet et al., 2019; Trinh et al., 2021). Here, we compare the performance of different commonly used fit algorithms by using simulated and experimental data, and we derive an analytic expression for their theoretical limits. With experimental data, we analyze the impact of sample inhomogeneity (intrinsic fluorescence lifetime variation of dye molecules) on obtained lifetime distributions, and we finally demonstrate that pattern-matching algorithms can be much more efficient than full lifetime-fitting in lifetime-based multiplexing.

## 2 Theory of Lifetime Determination

In a TCSPC lifetime measurement, the sample is excited with a train of sufficiently short laser pulses (ca. 100 femtoseconds to few dozen picoseconds) with fixed inter-pulse time period *T* (repetition period). For each detected photon, the arrival time *t* with respect to the last excitation pulse is recorded. The fluorescence lifetime *τ* can then be directly estimated from these arrival times as the mean (or standard deviation) of these *t*-values. However, this is only exact for a background-free measurement and for sufficiently large values of the repetition period *T* (*T* ≫ *τ*). For a precise lifetime determination with background and finite *T*, photon detection events are aggregated according to their arrival times, yielding the so-called TCSPC histogram, which is then fitted with a suitable model. Most fluorophores show a mono-exponential fluorescent decay behavior, so that one used a mono-exponential decay function with single decay time for fitting the TCSPC histogram (Lakowicz, 2006). In that case, the probability *p* for a photon to be detected at the time *t* is given by

$$p\left(t\right)=\left(1-b\right)\frac{\exp\left(-t/\tau \right)}{\tau \left(1-\exp\left(-T/\tau \right)\right)}+\frac{b}{T}$$ (1)

where *b* is the relative background amplitude (constant background). Experimentally, photon arrival times are grouped into *K* discrete TCSPC time channels *t*_*i*_ of finite width Δ*t*. In modern TCSPC systems, this time resolution Δ*t* of measuring photon detection times is usually much smaller than both the lifetime *τ* and the width of the so-called instrument response function (IRF) *σ*_IRF_, which is the experimentally measured TCSPC histogram for an ideal sample with infinitely fast fluorescence decay time. Therefore, any error that may be introduced by the TCSPC channel width is negligible, and the probability to detect a photon within one TCSPC channel is given by

$$p_{i}=\Delta t\;\left(1-b\right)\frac{\exp\left(-t_{i}/\tau \right)}{\tau \left(1-\exp\left(-T/\tau \right)\right)}+\frac{b}{K}.$$ (2)

For a total number $\hat{N}$ of expected photons, the expectation value for each bin is then given by

$${\hat{m}}_{i}=\hat{N}p_{i}=\hat{N}\Delta t\;\left(1-b\right)\frac{\exp\left(-t_{i}/\tau \right)}{\tau \left(1-\exp\left(-T/\tau \right)\right)}+\hat{N}\frac{b}{K}.$$ (3)

Here, ${\hat{m}}_{i}$ denotes the expected number of photons falling into the *i*th detection channel. It is important to note that the above equation is only correct for an infinitely narrow, delta-function like IRF, or when considering only TCSPC channels after a cut off of the part containing the IRF (TCSPC histogram starting some time *t*_cut_ after the peak of the IRF). This cut off eliminates the impact of the IRF on a TCSPC histogram and is a common approach when working with IRFs sufficiently narrow compared to the fluorescence lifetime. The values of *τ*, *b*, and, depending on the method, $\hat{N}$ are fitted by minimizing a suitable score function. Table 1 summarizes the defined symbols.

**TABLE 1:** Definitions of frequently used symbols.

Parameter	Description
*τ*	fluorescence lifetime
*b*	background fraction
*N*	total number of photons
*T*	repetition period
*t*	time since last pulse
*K*	number of TCSPC time bins
Δ*t*	width of TCSPC time bins
*p*	photon detection probability
*m*_*i*_	counts in time bin *i*
${\hat{m}}_{i}$	expected counts in time bin *i*
$\hat{N}$	expected total number of counts
*t*_cut_	cut-off time for tail-fits
$\chi_{\text{LS}}^{2}$	least square error
*ν*	degrees of freedom (here, *K* − 3)
*λ*	negative log-likelihood
$\sigma_{\tau }^{2}$	lifetime uncertainty (CRLB)
*t*_0_, *ρ*, *κ*	parameters of model IRF

### 2.1 Least-Square Estimators

The default score function for curve fitting with unknown error distribution is the sum of least-squares, i.e. the sum of the squared difference between data and estimate (L2-norm):

$$\chi_{\text{LS}}^{2}=\overset{K}{\underset{i=1}{\sum }}{\left({\hat{m}}_{i}-m_{i}\right)}^{2}$$ (4)

For single-photon detection, the number *m*_*i*_ of detected photons in channel *i* follows a Poissonian statistics, so that its variance is equal to its mean value (expectation value). In a weighted least-square minimization, each value in the *χ* ^2^-sum is weighted by the inverse of its variance, which requires to estimate, from the experimental data, the value of this variance. Pearson’s *χ* ^2^ used the model-fitted values ${\hat{m}}_{i}$ as an estimate for the variance, which leads to

$$\chi_{\text{P}}^{2}=\overset{K}{\underset{i=1}{\sum }}\frac{{\left({\hat{m}}_{i}-m_{i}\right)}^{2}}{{\hat{m}}_{i}}.$$ (5)

In contrast, Neyman’s *χ* ^2^ directly uses the experimentally measured values *m*_*i*_ as an estimate of the variance,

$$\chi_{\text{N}}^{2}=\overset{K}{\underset{i=1}{\sum }}\frac{{\left({\hat{m}}_{i}-m_{i}\right)}^{2}}{m_{i}}.$$ (6)

However, this expression becomes infinite whenever one of the values *m*_*i*_ becomes zero. Therefore, the denominator is either set to one in these cases ( $\chi_{N1}^{2}$ ), or the sum skips all *i* where *m*_*i*_ = 0 ( $\chi_{N2}^{2}$ ). In this work, we exclusively use $\chi_{N1}^{2}$ , because we observed that $\chi_{N2}^{2}$ leads to unstable fit results.

### 2.2 Maximum Likelihood Estimator

Unlike measurement in bulk or on densely labeled structures, single molecule measurements are always limited by the number of detected photons. Especially for low photon count numbers, the variance of these numbers significantly deviates from a Gaussian distribution which is, however, the basic assumption behind all least-square estimators. A maximum likelihood estimator (MLE) solves this problem by calculating the probability that a given set of parameters leads to an experimentally measured photon detection distribution. When assuming that the probability of detecting a photon in the *i*th channel of a TCSPC histogram is *p*_*i*_, then the likelihood of measuring a TCSPC histogram {*m*_*i*_} is given by (Baker and Cousins, 1984)

$$L=N!\overset{K}{\underset{i=1}{\prod }}\frac{{\left(p_{i}\right)}^{m_{i}}}{m_{i}!}.$$ (7)

This likelihood function takes extremely small values that are numerically difficult to handle and not very practical for comparing different parameter sets. To facilitate computation, constant factors are neglected, and one uses the negative logarithm of *L* instead of *L* itself. This leads to the negative log-likelihood function *λ* defined by

$$\lambda =-\overset{K}{\underset{i=1}{\sum }}m_{i}\ln p_{i}.$$ (8)

A similar estimator is the Poisson deviance which is derived from the likelihood ratio and relies on the estimated (fitted) values ${\hat{m}}_{i}$ instead of the probability (Baker and Cousins, 1984):

$$\chi_{\lambda }^{2}=2\overset{K}{\underset{i=1}{\sum }}\left(m_{i}\ln\frac{m_{i}}{\hat{m_{i}}}-\left(m_{i}-{\hat{m}}_{i}\right)\right)$$ (9)

By minimizing *λ*, the estimated number of photons $\hat{N}$ is implicitly fixed to the detected number of photons *N*. When replacing ${\hat{m}}_{i}=\hat{N}p_{i}$ and fixing $\hat{N}=N$ , Eq. 9 becomes Eq. 8 with a constant offset.

In the limit of high photon detection numbers *N*, both weighted least-square methods as well as the MLE give similar results (Bajzer et al., 1991).

### 2.3 Goodness of Fit

A widely used parameter for estimating the goodness of a fit is the reduced *χ* ^2^/*ν* with degrees of freedom *ν* = *K*—3 (minus three because we have the three fit parameters *τ*, *b*, and $\hat{N}$ ). For a perfect fit, *χ* ^2^/*ν* should be close to one. Smaller values indicate over-fitting, which is in the case of a mono-exponential model unlikely, and larger values indicate that the model does not describe the data completely. Both Pearson’s $\chi_{\text{P}}^{2}$ and the MLE $\chi_{\lambda }^{2}$ asymptotically approach the *χ* ^2^ distribution. However, for low number of counts per time bin ( ${\left\langle m_{i}\right\rangle }_{i}≲1$ ), the expectation value of $\chi_{\lambda }^{2}/\nu$ deviates from the value one while the expectation value of $\chi_{\text{P}}^{2}/\nu$ stays close to one at the cost of an increased variance. In practice, an increased variance is usually preferable over a count-dependent expectation value. The bias of the expectation value of $\chi_{\lambda }^{2}/\nu$ can be reduced by grouping adjacent time bins and thus decreasing the time resolution.

### 2.4 Lifetime Uncertainty

The Cramér-Rao lower bound (CRLB) uses the Fisher information of a measurement to calculate a lowest bound for the variance that an unbiased estimator can have. The amount of information conveyed by a measurement is shared between all unknown parameters ***θ***. For a mono-exponential decay with *N* photons and the probability distribution *p*(*t*) of Eq. 1, the Fisher matrix is given by:

$${\left[I\left(\theta \right)\right]}_{j,k}=\int_{0}^{T}\frac{\partial \ln\left(Np\left(t\right)\right)}{\partial \theta_{j}}\frac{\partial \ln\left(Np\left(t\right)\right)}{\partial \theta_{k}}Np\left(t\right)\;dt$$ (10)

The CRLB for each parameter is then given by the corresponding diagonal element of the inverse Fisher matrix:

$$\sigma_{j}^{2}={\left[I{\left(\theta \right)}^{-1}\right]}_{jj}$$ (11)

For TCSPC-measurements, typically both the lifetime and background need to be estimated: ***θ*** = {*τ*, *b*}. As discussed by Köllner and Wolfrum (1992), an unknown number of photons does not affect the uncertainty as off-diagonal elements $I_{N,k\neq N}$ become zero. A step-by-step derivation of the CRLB $\sigma_{\tau }^{2}$ for the lifetime is provided in the supplemental information.

### 2.5 Pattern Matching

Pattern matching is an alternative to lifetime fitting when the core task is to determine to which species a detected molecule belongs, among a discrete number of different species. Unlike lifetime fitting, pattern matching does not make any assumptions about the shape of the decay, and the only prerequisite is that reference decays of the separate species are available. To identify the most likely species to which a molecule belongs, the different negative log-likelihood values *λ*_*α*_ are calculated according to Eq. 8 by setting {*p*_*i*_} equal to the normalized probability distributions {*p*_*i*,*α*_} for each species *α*. The species with the lowest value of *λ* is then chosen as the most likely species. The rate of misidentifications depends on the number of photons *N* and the similarity of the patterns, see discussion in Enderlein and Sauer (2001) for details. The relative probability *f*_*α*_ for species *α* among a total of *S* species is given by

$$f_{\alpha }=\frac{\exp\left(-\lambda_{\alpha }\right)}{\sum_{\beta =1}^{S}\exp\left(-\lambda_{\beta }\right)}.$$ (12)

This equation is useful for rejecting molecules that cannot be classified with a high probability (Thiele et al., 2020). The relative probability is equivalent to the posterior probability of a Bayesian model comparison when assuming equal prior probabilities. In contrast to likelihoods or Bayes factors, the posterior probability can be averaged over multiple molecules or many time points.

Unlike the situation in usual fitting, the {*p*_*i*,*α*_} are the same for all TCSPC histograms. Therefore, the logarithms can be calculated in advance, and Eq. 8 can be implemented as a simple matrix multiplication. For this purpose, first the *K* × *S*-dimensional pattern matrix **P**_ln _ is calculated which contains the logarithm of the normalized patterns as row vectors. Second, the negative log-likelihood matrix **Λ** is obtained by multiplication with matrix **M**, which is the *J* × *K*-dimensional matrix constructed from the *J* TCSPC histograms (column vectors):

$$\Lambda =-M\cdot P_{\ln}$$ (13)

The resulting *J* × *S*-dimensional matrix **Λ** with entries *λ*_*j*,*α*_ allows for a fast calculation of the relative probabilities *f*_*j*,*α*_ with Eq. 12, or to directly determine the most likely pattern *x*_*j*_ for each TCSPC histogram with

$$x_{j}=\underset{\alpha }{\arg}\;\min\;\lambda_{j,\alpha }.$$ (14)

The *a priori* calculation of **P**_ln_, together with the single matrix multiplication step, enables efficient calculation of *λ*_*j*,*α*_ for thousands of TCSPC histograms in parallel and for many species. By employing a library of calculated decays {*p*_*i*,*α*_}, this approach allows for quick determination of the most likely parameter set using a grid-based search. We provide example code of pattern matching for classification and as well as for grid-based fitting in the supplementary material (Thiele, 2021b).

### 2.6 Fitting Using the Instrument Response Function

For lifetime values similar or shorter than the width of the IRF, it can be necessary to explicitly take the shape of the IRF into account. This is achieved by convolving the probability distribution {*p*_*i*_} (1) or expectation values $\left\{{\hat{m}}_{i}\right\}$ (Eq. 3) with the normalized IRF {*q*_*i*_}:

$${\hat{p}}_{i}^{*}={\hat{p}}_{i}⊛q_{i},\;{\hat{m}}_{i}^{*}={\hat{m}}_{i}⊛q_{i}$$ (15)

Here, ⊛ denotes a discrete, circular convolution. Subsequently, the score function is minimized with the convolved probability distributions $\left\{{\hat{p}}_{i}^{*}\right\}$ or expectation values $\left\{{\hat{m}}_{i}^{*}\right\}$ , respectively. This can be either performed for the tail of the decay only, or, more commonly, for the entire decay curve.

The IRF can be measured experimentally, or it can be approximated with a model. Typically, a Gaussian distribution or a shifted Gamma distribution, which reflects a potential asymmetry of the IRF, are used as parametric models. In this work, we employed a shifted Gamma distribution of the form

$$q_{i}=\left\{\begin{array}{cc} 0\; & t_{i}\leq t_{0}\\ \Delta t\kappa^{\rho }{\left(t_{i}-t_{0}\right)}^{\rho -1}\exp\left[-\kappa \left(t_{i}-t_{0}\right)\right]/\Gamma \left(\rho \right)\; & t_{i}>t_{0} \end{array}\right.$$ (16)

where the distribution depends on the following three parameters: starting time of the peak *t*_0_, shape parameter *ρ*, and rate parameter *κ*.

## 3 Methods

### 3.1 Simulations

The different least-square estimators and the maximum-likelihood estimator were tested on simulated data. If not stated otherwise, the following parameters were used: lifetime *τ* = 2 ns, background *b* = 0.2, repetition period *T* = 25 ns, TCSPC time resolution Δ*t* = 0.016 ns. Using these parameters and the average total number *N* of detected photons, the expectation values $\left\{{\hat{m}}_{i}\right\}$ were calculated for each time bin following Eq. 3. To generate a simulated decay {*m*_*i*_}, Poisson-distributed random variables with expectation value $\left\{{\hat{m}}_{i}\right\}$ were drawn. The simulated data was fitted with the model function (Eq. 3) by minimizing each estimator (Eqs 5, 6 and 8) with a Nelder-Mead simplex algorithm. Initial fit values were calculated by multiplying the true value with a random number between 0.5 and 1.5 to obtain a low-precision initial guess value. Simulation and fitting was repeated 10^5^ times to obtain a sufficiently large distribution of fit results. The simulation was implemented in Matlab (R2020a, The MathWorks Inc.).

To investigate the influence of the IRF on the fitted lifetime, a dedicated simulation was performed. First, the experimental IRF of the confocal microscope described in (Thiele et al., 2020) was determined by measuring backscattering from a coverglass coated with a 10 nm gold film. The substrate preparation is described in detail in (Ghosh et al., 2021). A normalized experimental IRF is obtained from the measured TCSPC histogram by subtracting the background, defined as the average count level in the second half of the TCSPC histogram, and dividing by its sum. A parametric IRF was obtained by fitting the TCSPC histogram with (1 − *b*)*q*_*i*_ + *b*/*K*, where *q*_*i*_ is defined as in Eq. 16, by minimizing the negative log-likelihood (Eq. 8) for the parameters *t*_0_, *ρ*, *κ*, and *b* with a Nelder-Mead simplex algorithm. Subsequently, the parametric IRF was calculated with these *t*_0_, *ρ*, and *κ*.

Similar to the previous simulation, a decay with background *b* = 0.2, repetition period *T* = 25 ns, and time resolution Δ*t* = 0.016 ns was calculated, while its lifetime was varied from 0.025 ns to 2.0 ns in 0.025 ns increments. The calculated decay was convolved with the experimental IRF, and 10^5^ TCSPC histograms with a mean value of 2000 photons were simulated. From the TCSPC histograms, the lifetime values were determined by an MLE grid search based on the pattern matching described in Section 2.4 with 500 lifetime values linearly spaced from 0.01 ns to 3.00 ns and 60 different background values. The reference decays were calculated in three different ways: (1) mono-exponential decay without IRF, (2) mono-exponential decay convolved with the experimental IRF, and mono-exponential decay convolved with the parametric IRF. For case (1), the likelihood was calculated using the tail of the decay starting *t*_cut_ = 0.2 ns after the maximum of the sum of all decays. The correspondingly shortened reference decays were normalized prior to calculating the likelihood values. For case (2) and (3), the likelihood was calculated with the entire TCSPC histogram.

### 3.2 Experimental Data

For checking the different lifetime-fitting approaches on real experimental data, we used dSTORM images of three different structures: Alexa 647-labeled microtubules, Atto 655-labeled clathrin pits in fixed COS7 cells, and 3 µm polystyrene beads decorated with Alexa 647-labeled DNA. All experimental data were taken from (Thiele et al., 2020), where details on the sample preparation and measurement can be found. The data was processed with TrackNTrace (see below) to extract single-molecule TCSPC histograms. The tail of the single-molecule decay curves, starting 0.2 ns after the maximum in the sum of all decays, were fitted by minimizing the negative log-likelihood function (Eq. 8) with a Nelder-Mead simplex algorithm. Initial fit values were determined by using a pattern matching of the decay curves. For this purpose, 500 lifetime values linearly spaced from 0.01 to 5.00 ns and 60 different background values were used.

For the analysis of the experimentally obtained lifetime distributions (Figure 3), the single molecule data was sorted according to the number of photons per TCSPC histogram, and then divided into 30 equally-sized groups. Only molecules with an image size (standard deviation) between 100 and 180 nm, with at least 25 photons in the TCSPC histogram, and with a reduced Pearson’s $\chi_{\text{P}}^{2}/\nu$ of their lifetime fit between 0.8 and 1.2 were included in the final analysis.

For each group, the standard deviation *σ* of the single molecule lifetimes and the analytic CRLB based on the average number of photons ⟨*N*⟩, the lifetime ⟨*τ*⟩ and the background ⟨*b*⟩, was calculated. The width of the sample-intrinsic lifetime variation *σ*_sample_ was determined by fitting the *σ* for ⟨*N*⟩ > 100 with an decay of the form *σ* = *a*⟨*N*⟩^−*k*^ + *σ*_sample_ with empirical fit parameters *a*, *k*, and intrinsic sample-related variance *σ*_sample_ by minimizing $\chi_{\text{LS}}^{2}$ with a Nelder-Mead simplex algorithm.

For exemplifying and checking the pattern-matching algorithm, we used data obtained from COS7 cells labeled with either Alexa 647 or Atto 655, from which synthetic data with mixed labeling were generated. Only molecules with an image size between 100 and 180 nm and at least 50 photons in the TCSPC histogram were used for further analysis. As reference patterns, we used normalized decay curves of pure samples of Alexa 647 and Atto 655, respectively. With these reference patterns, the relative probability that a localized molecule was either Alexa 647 or Atto 655 was calculated, following Eq. 12. Based on these relative probabilities and the known identities of the samples, the receiver operating characteristic (ROC) and the area under curve (AUC) were calculated. As comparison, the same curves were calculated using MLE fitted lifetime values as classification score.

## 4 Results

### 4.1 Simulations and Analytical Results

The CRLB yields the minimum variance that can be attained by an unbiased estimator. For a mono-exponential decay with background and an infinite time resolution, an analytical expression for the CRLB can be given. It has recently been shown numerically that the TCSPC channel discretization does not considerably affect the CRLB as long as the time bins are much narrower than the lifetime (Trinh et al., 2021). In contrast, background substantially affects a CRLB (Köllner and Wolfrum, 1992). The full expression of the CRLB for the variance $\sigma_{\tau }^{2}$ of the lifetime *τ* as function of *τ* itself, the number of photons *N*, the background fraction *b*, and the repetition period *T*, is given in the supplementary information (see Supplementary Equation S1). It is, however, useful to analyze some limiting cases. In the limiting case of an infinitely large repetition period *T*, the estimator is only limited by Poisson noise:

$$\underset{T\to \infty }{\lim}\sigma_{\tau }^{2}=\frac{\tau^{2}}{N}\frac{1}{1-b}$$ (17)

Here, *N*(1 − *b*) photons correspond to the fluorescence signal while the *Nb* background photons do not carry any lifetime information. Since the off-diagonal elements of the Fisher matrix vanish ( ${\left[I\left(\theta \right)\right]}_{\tau ,b}=0$ ) for *T* → *∞*, the knowledge of the background does not affect the result. For a finite value of *T*, these off-diagonal elements become non-zero and do increase the value of $\sigma_{\tau }^{2}$ . Therefore, it makes a difference whether the background level needs to be estimated independently as an additional fit parameter, or whether it is known in advance. If it is known that the background is zero one finds

$$\underset{b\to 0}{\lim}\sigma_{\tau }^{2}=\frac{\tau^{2}}{N}\frac{2\left(1-\cosh\left(\chi \right)\right)}{2+\chi^{2}-2\cosh\left(\chi \right)}\;\text{with}\;\chi =\frac{T}{\tau }$$ (18)

while for an unknown but zero background one finds the generally larger value

$$\underset{b\to 0}{\lim}\sigma_{\tau }^{2}=\frac{\tau^{2}}{N}\frac{4\sinh\left(\frac{\chi }{2}\right)\left(\chi^{2}-2\cosh\left(\chi \right)+2\right)}{\left(\chi^{4}+12\chi^{2}+12\right)\sinh\left(\frac{\chi }{2}\right)-4\chi^{3}\cosh\left(\frac{\chi }{2}\right)-4\sinh\left(\frac{3\chi }{2}\right)}.$$ (19)

A comparison between known and unknown background levels and calculations for different combinations of background and repetition period *T* are provided in Supplementary Figure S1.

**FIGURE 1:**
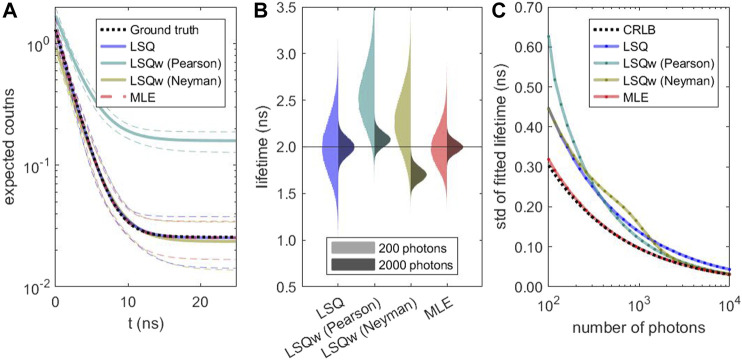
Performance of different lifetime estimators: Based on a calculated decay with 2 ns lifetime and 20% background photons, 10^5^ decay curves with Poissonian noise were simulated for different numbers of photons and fitted using different estimators. **(A)** Ground truth (black) and fit results of simulated decays with 200 photons (expectation value). The thick, colored lines indicate the median, and the thin, dashed lines the 5 and 95% quantiles. **(B)** Distribution of fitted lifetime values for decays with 200 photons **(left half)** and 2000 photons **(right half)**. **(C)** Standard deviation of the lifetime distributions and calculated CRLB as a function of the number of photons in the decay.

To validate the performance of the different estimators, simulated TCSPC histograms were fitted and the fit results compared to the ground truth. Especially at low photon counts, there is a striking difference between the estimators. In Figure 1A, it is apparent that weighted least-square estimators are biased and do not reproduce the correct decay curve. The distribution of fitted lifetime values (Figure 1B) emphasizes this count-dependent bias. In contrast, the median lifetime value recovered by the unweighted LSQ and the MLE are very close to the ground truth. However, the MLE achieves this with much less uncertainty. The distribution of the fitted number of photons, background level, and bias of median lifetime values in dependence of the number of counts are given in Supplementary Figure S2. As shown in Figure 1C, the standard deviation of the lifetime values obtained with weighted LSQ and MLE do approach the theoretical limit of the CRLB. However, solely MLE provides a performance close to the CRLB with no substantial bias of the lifetime values, even for photon detection numbers of only a few hundred. This behavior is not specific for the chosen simulation parameters. In the supplementary material, see Supplementary Table S1, we present additional simulations for lower lifetimes, for shorter repetition rates, and for higher and lower background values, confirming our results described above. In good agreement with our results, Santra et al. (2016) found no bias for MLE but substantial bias for least-square estimators when fitting solution measurements and neglecting background.

#### 4.1.1 Influence of the Instrument Response Function

**FIGURE 2:**
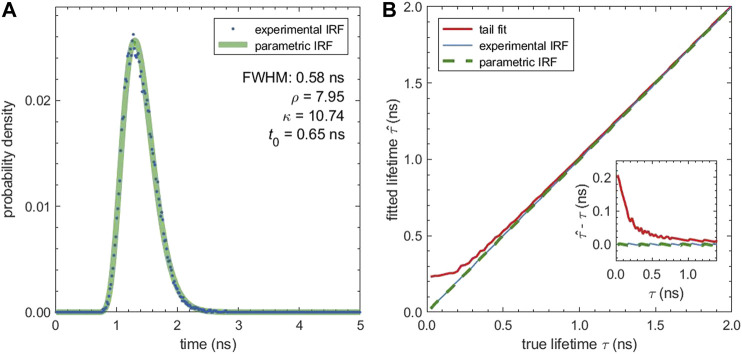
Influence of a non-ideal IRF on the fitted lifetime: **(A)** Experimental IRF measured from gold scattering (blue dots) and parametric IRF (green line). The parametric IRF is described by a shifted Gamma distribution with the parameters given in the plot. **(B)** Median fitted lifetime for simulated decays with the experimental IRF and different lifetimes. The fitted lifetime was determined by fitting the tail of the decays with a mono-exponential function (tail fit) and by fitting the entire decay curve using either the experimental IRF or the parametric IRF. The inset shows the absolute difference between the fitted and the true lifetime in dependence of the true lifetime. All fits are grid-based MLE fits, and tail fits use a cut off *t*_cut_ = 0.2 ns.

The simulations so far neglected the influence of the IRF which is equivalent to assuming a dirac-like IRF. In reality, the IRF has a finite width and can influence the fitted lifetime. Figure 2 shows an experimental IRF obtained by recording the back-scattered light form a gold-covered coverslip, together with its parametric fit. The parametric IRF was obtained by fitting the scattering measurement and has a full width at half maximum (FWHM) of 0.58 ns. The good fit quality confirms that the shifted Gamma distribution is an excellent model for the true IRF.

To evaluate the effect of the IRF, we simulated TCSPC measurements using the experimental IRF and for sample lifetimes (ground-truth) between 0.025 and 2.0 ns. The computed TCSPC curves were then fitted with and without IRF using an MLE grid search. Figure 2B shows that the tail fit leads to a bias towards larger lifetime values when the actual lifetime comes closer to the width of the IRF. For lifetime values close to zero, the bias reaches 0.2 ns. This bias can be eliminated by taking the IRF explicitly into account. Fits with the IRF, both with the experimental IRF (which was used for the TCSPC simulation) as well as with its parametric form, lead to negligible bias for all tested lifetimes.

With a FWHM of above 0.5 ns, the IRF we used for the simulation was rather broad due to the employed diode laser. For a narrower IRF, e.g. with a typical white light laser, the influence of the IRF will be even less pronounced and the bias of tail-fit results reduced.

### 4.2 Experimental Results

#### 4.2.1 Estimation of the Intrinsic Lifetime Distribution

**FIGURE 3:**
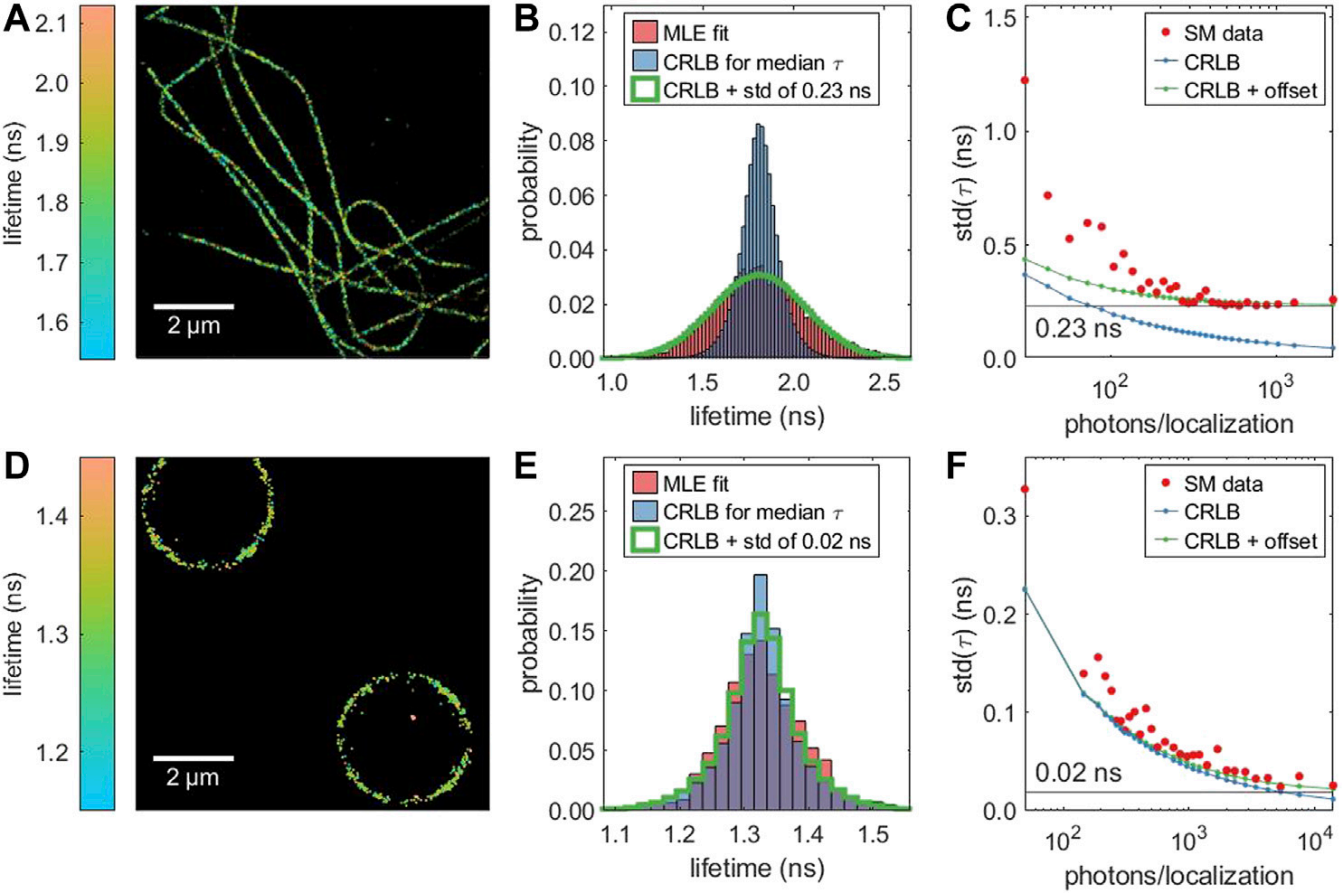
Lifetime-resolved dSTORM of two different samples with Alexa 647: **(A, B, C)** immunostained microtubules in fixed COS7 cells and **(D, E, F)** DNA-functionalized micro-beads. **(A,D)** Super-resolved FL-SMLM image. **(B, E)** Distribution of the single molecule lifetime values (red), CRLB-limited distribution assuming equal lifetime values for all molecules, and CRLB-limited distribution with an additional broadening. **(C, F)** Dependence of the standard deviation of the fitted lifetime on the number of photons. With an increasing number of photons, the standard deviation (red) approaches a limit which is caused by sample inhomogeneity (intrinsic variation of lifetime values). In contrast, the corresponding CRLB (blue) approaches zero. The green line represents the square root of the sum of the variance as predicted by the CRLB and the intrinsic variance of the sample.

In real measurements, single-molecule lifetime values are not only affected by Poisson noise but also by intrinsic sample inhomogeneity (variation of intrinsic lifetime values). Figure 3 compares two samples: Alexa 647 in a cellular environment exhibits a broader lifetime distribution that cannot be explained solely by the CRLB, while the lifetime distribution of DNA-conjugated Alexa 647 on a polymer bead surface is close to the CRLB. Unlike the CRLB, this sample-dependent contribution to the lifetime variance does not dependent on the number of detected photons. Therefore, it can be estimated as the asymptotic limit of the standard deviation of the lifetimes as a function of the number of detected photons, as shown in Figures 3C,F. When taking this additional intrinsic sample-related variance into account, the theoretical estimate (green line in Figures 3B,E) does closely match the measured lifetime distribution. By simulating a sample with a known lifetime inhomogeneity (Supplementary Figure S13), we can confirm this method recovers the intrinsic variance. In the cellular environment, we observe a small dependence of the average lifetime on photon number (Supplementary Figure S14). This adds to the overall width of the lifetime distribution (Figure 3B), but does not affect our estimation of the intrinsic variance as the standard deviation is independent of the mean.

The clear difference between the average lifetimes in the two different samples matches results in the literature that the lifetime of cyanines like Alexa 647 sensitively depends on the environment (Buschmann et al., 2003; Klehs et al., 2014).

**FIGURE 4:**
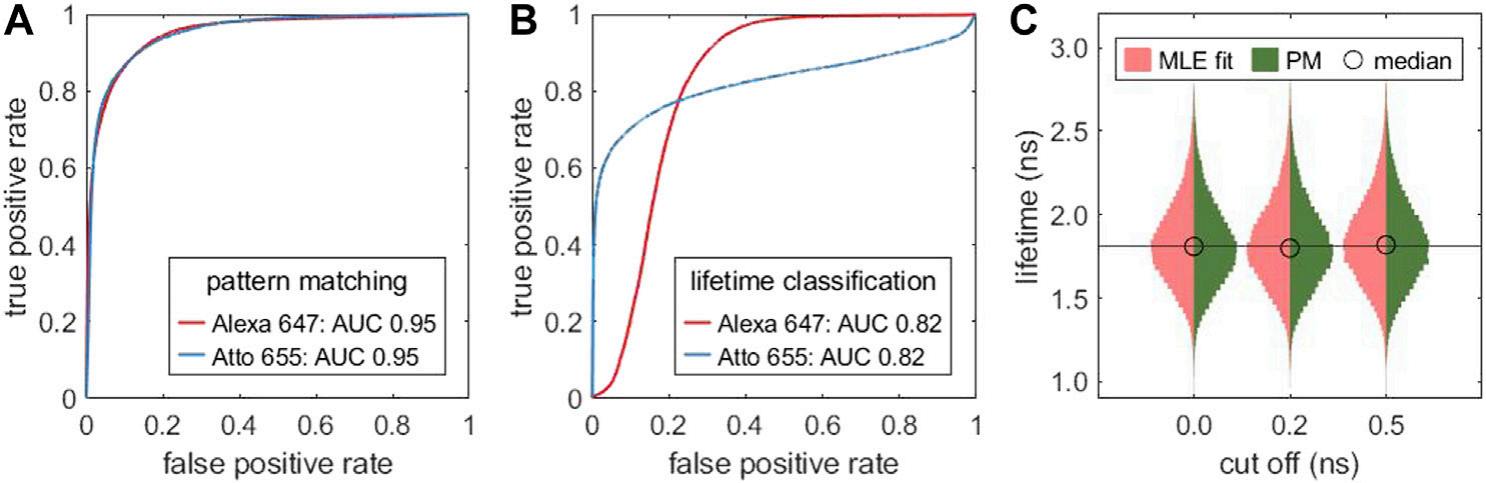
Pattern matching as alternative to lifetime fitting: Receiver operating characteristic (ROC) curve for classifying a mixture of two different fluorophores (Alexa 647, Atto 655) with different lifetimes. In **(A)**, the classification is based on pattern matching, in **(B)** on the MLE fitted single molecule lifetimes. The data was generate by combining measurements of two samples, each labeled with one fluorophore. **(C)** Comparison of the lifetime distribution obtained by MLE lifetime fitting (red) and an MLE grid search based on pattern matching (dark green) for different cut off values *t*_cut_ relative to the TCSPC maximum. Based on the same measurement as Figure 3A.

For experimental data, all lifetimes in this work are determined with mono-exponential tail-fits. The interval of the TCSPC histogram which is used for lifetime fitting starts at or short time (cut off *t*_cut_) after the maximum of the TCSPC histogram. Figure 4C illustrates that the cut off does not notably affect the resulting lifetime distribution for a mono-exponential sample.

#### 4.2.2 Pattern Matching

A pattern matching compares the TCSPC histogram with a library of reference patterns. This allows one to classify single molecules without fitting their lifetime and setting a lifetime threshold. Figure 4 compares the performance of pattern matching (A) with lifetime-fitting based classification (B). Pattern matching offers a higher sensitivity and specificity which is reflected in the larger area under the curve (AUC) as compared to lifetime-fitting based classification. The reference patterns, shown in Supplementary Figure S15A, reveal that the decay curves are not strictly mono-exponential. Therefore, overlapping lifetime distributions (Supplementary Figure S15B) can be separated better by pattern matching than by lifetime fitting. The specificity can be improved by removing molecules with low number of photons.

Pattern matching is also useful for quickly finding good guess values over a limited parameter space using a parallel grid search. Figure 4C shows that the lifetimes obtained by pattern matching closely resemble the distribution obtained from MLE fitting. For highest precision, the lifetime values could be refined with a subsequent precise MLE fit.

## 5 Discussion and Conclusion

Using Monte-Carlo simulations, we have demonstrated that MLE-based fitting outperforms common LSQ-based fitting and achieves close to shot-noise limited accuracy. An analytic expression for this limit, the CRLB for a mono-exponential decay with unknown background and finite repetition period was derived. In SMLM, the localization uncertainty derived from the CRLB has become an indispensable parameter for data filtering. We suggest to use the lifetime uncertainty in a similar fashion for filtering lifetime-resolved single-molecule data, to improve separation between different species or states, and to estimate experimental limitations. To facilitate its application, we provide a Python implementation of the CRLB calculation (Thiele, 2021a).

Our simulations confirmed that the fit uncertainties originating from photon statistics, background, and finite repetition period can be well estimated by the CRLB. However, in actual single-molecule lifetime experiments, additional sources of uncertainty need to be considered. Many fluorophores are known to be sensitive to their local environment. This can be a desired effect, e.g. for lifetime-based environmental sensing (Klymchenko, 2017). However, for many applications, including lifetime-based multiplexing or FRET, the intrinsic lifetime variation should be as narrow as possible because any broadening increases final uncertainties of the results. Therefore, it is important to quantify the intrinsic lifetime variation across a single species of labels/dyes which is, unlike the CRLB, independent on the number of detected photons. We have shown that this intrinsic lifetime variation can be extracted from the photon-number dependence of the experimentally determined lifetime distribution. As presented in Figure 3, the same fluorophore but in different samples can show very different lifetime variation, with measurements in cells exhibiting a considerably broader variation. This suggests that single-molecule lifetime measurements in cells might not always be limited only by the instrument response function or the photon statistics, but can be also limited by intrinsic lifetime variations of a sample itself.

In FLIM experiments, the fluorescence lifetime is often used to identify different states, e.g. high or low FRET efficiency, or distinguish between different species with different lifetimes. FL-SMLM extracts this information from single molecules and allows for a discrete classification of imaged molecules based on their lifetimes. For such an application, full lifetime fitting is not always the most efficient way for classification. As demonstrated in Figure 4, pattern matching outperforms lifetime-based classification. Pattern matching compares a measured TCSPC histogram with reference histograms, which can be done extremely fast, and which does not require any specific knowledge about the character of a fluorescence decay (e.g. mono-exponential decay) while utilizing all photons of a TCSPC histogram. When using a large number of calculated decays, pattern matching can serve as a way of unbiased lifetime estimation within a fixed parameter space. Since this is very fast, it is attractive for initial parameter guesses and might be useful when many thousands of lifetimes need to be determined, e.g. in pixel-wise time-resolved data.

To facilitate the analysis of FL-SMLM data, we have amended our open source software package TrackNTrace (Stein and Thiart, 2016) with the ability to extract TCSPC histograms and to fit lifetime values. It conveniently covers all required computational steps, from reading raw data to reconstructing FLIM images, all within a single GUI-based app. In this app, lifetime values can be determined with fast pattern matching and, optionally, by subsequent refinement with precise MLE-based fitting.

## Data Availability

Publicly available datasets were analyzed in this study. This data can be found here: https://projects.gwdg.de/projects/cfl-smlm/repository. The supplemental scripts are available at https://doi.org/10.5281/zenodo.5093591 and example code for pattern matching at https://doi.org/10.5281/zenodo.5423457. Our analysis software TrackNTrace can be found at https://github.com/scstein/TrackNTrace.
