# Supplementary material for: Advanced Data Analysis for Fluorescence-Lifetime Single-Molecule Localization Microscopy
Source: Front Bioinform. 2021 Nov 19;1:740281. doi: 10.3389/fbinf.2021.740281 (PMC9581058; doi:10.3389/fbinf.2021.740281)
Supplement: Supplementary file 1 [file DataSheet2.PDF]

Derivation of the CRLB

Probability distribution

We assume a continues, mono-exponential probability distribution p with a constant background. The probability distribution is normalised over the range 0 to T. Therefore, the expectation value is given by $n\,p$.

The following variables are used:

t: time

τ: fluorescence lifetime

T: pulse repetition period/measurement window

b: background fraction ($b = 0$, no background; $b = 1$ only background)

n: number of photons in decay

In[1]:=
```
pAssumptions = { t ≥ 0 , T > 0, τ > 0, 1 > b ≥ 0, n > 0, χ > 0, {t, T, τ, b, n, χ} ∈ Reals};
p = FullSimplify[ (1 - b) *
     PDF[ExponentialDistribution[1 / τ], t] / CDF[ExponentialDistribution[1 / τ], T] +
   b / T, pAssumptions, ExcludedForms → {Exp[_]}]
```

Out[2]=
$$\frac{b}{T} + \frac{(-1+b)\, e^{-\frac{t}{\tau}}}{\left(-1+e^{-\frac{T}{\tau}}\right)\tau}$$

Check normalization:

In[3]:=
$$\int_0^T p \, dt$$

Out[3]= 1

CRLB for a known background

In this case only the lifetime needs to be estimated ($\theta = \{\tau\}$) and the Fisher matrix f has just one entry.

In[4]:=
```
fττ = Integrate[(∂τ Log[n * p])^2 * (n * p), {t, 0, T}, Assumptions → pAssumptions]
```

Out[4]=
```
- ((n (b T^3 + e^(T/τ) T^3 - b e^(T/τ) T^3 + 2 b T^2 τ - 2 b e^(T/τ) T^2 τ - T τ^2 + 2 b T τ^2 + 2 e^(T/τ) T τ^2 - 4 b e^(T/τ) T τ^2 -
      e^(2T/τ) T τ^2 + 2 b e^(2T/τ) T τ^2 + b T^2 τ Log[(b (-1 + e^(T/τ)) τ) / (T - b T + b (-1 + e^(T/τ)) τ)] -
      2 b e^(T/τ) T^2 τ Log[(b (-1 + e^(T/τ)) τ) / (T - b T + b (-1 + e^(T/τ)) τ)] +
      b e^(2T/τ) T^2 τ Log[(b (-1 + e^(T/τ)) τ) / (T - b T + b (-1 + e^(T/τ)) τ)] -
      b T^2 τ Log[e^(T/τ) (T - b T + b (-1 + e^(T/τ)) τ)] - 2 b T τ^2 Log[e^(T/τ) (T - b T + b (-1 + e^(T/τ)) τ)] +
      2 b e^(T/τ) T τ^2 Log[e^(T/τ) (T - b T + b (-1 + e^(T/τ)) τ)] -
      b τ^3 Log[e^(T/τ) (T - b T + b (-1 + e^(T/τ)) τ)] + 2 b e^(T/τ) τ^3 Log[e^(T/τ) (T - b T + b (-1 + e^(T/τ)) τ)] -
      b e^(2T/τ) τ^3 Log[e^(T/τ) (T - b T + b (-1 + e^(T/τ)) τ)] +
      2 b T^2 τ Log[(T - b T + b (-1 + e^(T/τ)) τ) / (b (-1 + e^(T/τ)) τ)] -
      2 b e^(T/τ) T^2 τ Log[(T - b T + b (-1 + e^(T/τ)) τ) / (b (-1 + e^(T/τ)) τ)] +
      2 b T τ^2 Log[(T - b T + b (-1 + e^(T/τ)) τ) / (b (-1 + e^(T/τ)) τ)] -
      4 b e^(T/τ) T τ^2 Log[(T - b T + b (-1 + e^(T/τ)) τ) / (b (-1 + e^(T/τ)) τ)] +
      2 b e^(2T/τ) T τ^2 Log[(T - b T + b (-1 + e^(T/τ)) τ) / (b (-1 + e^(T/τ)) τ)] +
      b T^2 τ Log[-b τ + e^(T/τ) (T - b T + b τ)] + 2 b T τ^2 Log[-b τ + e^(T/τ) (T - b T + b τ)] -
      2 b e^(T/τ) T τ^2 Log[-b τ + e^(T/τ) (T - b T + b τ)] + b τ^3 Log[-b τ + e^(T/τ) (T - b T + b τ)] -
      2 b e^(T/τ) τ^3 Log[-b τ + e^(T/τ) (T - b T + b τ)] + b e^(2T/τ) τ^3 Log[-b τ + e^(T/τ) (T - b T + b τ)] -
      2 b (-1 + e^(T/τ)) τ^2 (T + τ - e^(T/τ) τ) PolyLog[2, ((-1 + b) e^(T/τ) T) / (b (-1 + e^(T/τ)) τ)] +
      2 b (-1 + e^(T/τ)) τ^2 (e^(T/τ) (T - τ) + τ) PolyLog[2, (T - b T) / (b τ - b e^(T/τ) τ)] -
      2 b τ^3 PolyLog[3, ((-1 + b) e^(T/τ) T) / (b (-1 + e^(T/τ)) τ)] +
      4 b e^(T/τ) τ^3 PolyLog[3, ((-1 + b) e^(T/τ) T) / (b (-1 + e^(T/τ)) τ)] -
      2 b e^(2T/τ) τ^3 PolyLog[3, ((-1 + b) e^(T/τ) T) / (b (-1 + e^(T/τ)) τ)] +
      2 b τ^3 PolyLog[3, (T - b T) / (b τ - b e^(T/τ) τ)] -
      4 b e^(T/τ) τ^3 PolyLog[3, (T - b T) / (b τ - b e^(T/τ) τ)] +
      2 b e^(2T/τ) τ^3 PolyLog[3, (T - b T) / (b τ - b e^(T/τ) τ)])) / ((-1 + e^(T/τ))^2 T τ^4))
```

Mathematica needs some help with the simplification : We need to reduce all Log expressions and collect the τ to enable cancelling them.
For efficient numerical evaluation, Powers are expanded again to avoid errors by to large arguments in the Log. The terms with PolyLog are grouped to minimise calls to PolyLog.

In[5]:=
```
SimplifyCRLB[exprs_] := Assuming[pAssumptions,
  Simplify[
   Simplify[exprs //. {Log[a_] + Log[b_] :→ Log[a * b], x_ * Log[a_] :→ Log[a^x],
       a_ * τ + b_ * τ :→ (a + b) * τ}] (* Cancel τ in the log terms *)
    //. {Log[a_ * b_^x_] :→ Log[a] + x * Log[b],
     a_ * PolyLog[x_, c_] + b_ * PolyLog[x_, c_] :→ (a + b) * PolyLog[x, c]}]
   (* Optimise for numerical evaluation *)
 ]
```

We substitute the repetition period with a relative repetition period: $\chi = T/\tau$

The CRLB (σ_τ^2) is given the inverse Fisher matrix:

```
In[6]:= στSQ = SimplifyCRLB[1 / fττ //. {T → χ τ}]

Out[6]= - (((-1 + e^χ)^2 τ^2 χ) /
     (n (-χ + 2 b χ + 2 e^χ χ - 4 b e^χ χ - e^(2 χ) χ + 2 b e^(2 χ) χ + 2 b χ^2 - 2 b e^χ χ^2 + b χ^3 + e^χ χ^3 - b e^χ χ^3 -
         2 b (-1 + e^χ) (-1 + e^χ - χ) χ (Log[b] + Log[-1 + e^χ] - Log[b (-1 + e^χ - χ) + χ]) +
         b (-1 + e^χ)^2 χ^2 (Log[b (-1 + e^χ)] - Log[b (-1 + e^χ - χ) + χ]) -
         b (1 - e^χ + χ)^2 Log[(e^χ (b (-1 + e^χ - χ) + χ)) / (-b + e^χ (b + χ - b χ))] +
         2 b (-1 + e^χ) (1 + e^χ (-1 + χ)) PolyLog[2, ((-1 + b) χ) / (b (-1 + e^χ))] +
         2 b (-1 + e^χ) (-1 + e^χ - χ) PolyLog[2, ((-1 + b) e^χ χ) / (b (-1 + e^χ))] +
         2 b (-1 + e^χ)^2 PolyLog[3, ((-1 + b) χ) / (b (-1 + e^χ))] -
         2 b (-1 + e^χ)^2 PolyLog[3, ((-1 + b) e^χ χ) / (b (-1 + e^χ))])))
```

Limit for zero background:

```
In[7]:= Assuming[pAssumptions, FullSimplify[1 / Limit[1 / στSQ, b → 0, Direction → "FromAbove"]]]
        (* The limits of 1/στSQ are somehow much faster to compute. *)

   ... Limit: Warning: Assumptions that involve the limit variable are ignored.

Out[7]= - ((2 τ^2 (-1 + Cosh[χ])) / (n (2 + χ^2 - 2 Cosh[χ])))
```

Limit for infinite pulse period:

```
In[8]:= 1 / Limit[1 / στSQ, χ → ∞, Assumptions → pAssumptions, Direction → "FromBelow"]

   ... Limit: Warning: Assumptions that involve the limit variable are ignored.

Out[8]= τ^2 / (n - b n)
```

CRLB for an unknown background

Now lifetime and background need to be estimated ($\theta = \{\tau, b\}$). The Fisher matrix has the form

$$f = \begin{pmatrix} f_{\tau\tau} & f_{\tau b} \\ f_{\tau b} & f_{bb} \end{pmatrix}.$$

Calculate $f_{\tau b}$ and f_{bb}, and assemble Fisher matrix.

In[9]:=
```
fτb =
 Integrate[(∂τ Log[n * p]) * (∂b Log[n * p]) * (n * p), {t, 0, T}, Assumptions → pAssumptions]
```

Out[9]=
```
-((n (2 T^2 - b T^2 + b ⅇ^(T/τ) T^2 + 2 T τ - 2 ⅇ^(T/τ) T τ - 2 T τ Log[ⅇ^(T/τ) (T - b T + b (-1 + ⅇ^(T/τ)) τ)] -
     2 τ^2 Log[ⅇ^(T/τ) (T - b T + b (-1 + ⅇ^(T/τ)) τ)] + 2 ⅇ^(T/τ) τ^2 Log[ⅇ^(T/τ) (T - b T + b (-1 + ⅇ^(T/τ)) τ)] +
     2 T τ Log[(T - b T + b (-1 + ⅇ^(T/τ)) τ) / (b (-1 + ⅇ^(T/τ)) τ)] -
     2 b T τ Log[(T - b T + b (-1 + ⅇ^(T/τ)) τ) / (b (-1 + ⅇ^(T/τ)) τ)] -
     2 ⅇ^(T/τ) T τ Log[(T - b T + b (-1 + ⅇ^(T/τ)) τ) / (b (-1 + ⅇ^(T/τ)) τ)] +
     2 b ⅇ^(T/τ) T τ Log[(T - b T + b (-1 + ⅇ^(T/τ)) τ) / (b (-1 + ⅇ^(T/τ)) τ)] +
     2 b T τ Log[1 - (b (-1 + ⅇ^(T/τ)) τ) / ((-1 + b) T)] - 2 b ⅇ^(T/τ) T τ
      Log[1 - (b (-1 + ⅇ^(T/τ)) τ) / ((-1 + b) T)] + 2 T τ Log[-b τ + ⅇ^(T/τ) (T - b T + b τ)] +
     2 τ^2 Log[-b τ + ⅇ^(T/τ) (T - b T + b τ)] - 2 ⅇ^(T/τ) τ^2 Log[-b τ + ⅇ^(T/τ) (T - b T + b τ)] +
     2 (-1 + b) (-1 + ⅇ^(T/τ)) τ^2 PolyLog[2, ((-1 + b) ⅇ^(T/τ) T) / (b (-1 + ⅇ^(T/τ)) τ)] -
     2 b (-1 + ⅇ^(T/τ)) τ^2 PolyLog[2, (b (-1 + ⅇ^(T/τ)) τ) / ((-1 + b) T)] -
     2 b τ^2 PolyLog[2, -((b (1 - ⅇ^(-T/τ)) τ) / (T - b T))] +
     2 b ⅇ^(T/τ) τ^2 PolyLog[2, -((b (1 - ⅇ^(-T/τ)) τ) / (T - b T))] - 2 τ^2
      PolyLog[2, (T - b T) / (b τ - b ⅇ^(T/τ) τ)] + 2 b τ^2 PolyLog[2, (T - b T) / (b τ - b ⅇ^(T/τ) τ)] +
     2 ⅇ^(T/τ) τ^2 PolyLog[2, (T - b T) / (b τ - b ⅇ^(T/τ) τ)] -
     2 b ⅇ^(T/τ) τ^2 PolyLog[2, (T - b T) / (b τ - b ⅇ^(T/τ) τ)])) / (2 (-1 + b) (-1 + ⅇ^(T/τ)) T τ^2))
```

In[10]:=
```
fbb = Integrate[(∂b Log[n * p])^2 * (n * p), {t, 0, T}, Assumptions → pAssumptions]
```

Out[10]=
```
(n (T - b T + τ Log[T - b T + b (-1 + ⅇ^(T/τ)) τ] - τ Log[-b τ + ⅇ^(T/τ) (T - b T + b τ)])) / ((-1 + b)^2 b T)
```

In[11]:=
```
f = {{fττ, fτb}, {fτb, fbb}};
```

The CRLB ($\sigma_\tau{}^2$) is given the first diagonal element of the inverse Fisher matrix:

In[12]:=
```
στSQ = SimplifyCRLB[First@First@Inverse[f //. {T → χ τ}]]
```

Out[12]=
```
-((4 (-1 + ⅇ^χ)^2 τ^2 χ (χ - b χ + Log[b (-1 + ⅇ^χ - χ) + χ] - Log[-b + ⅇ^χ (b + χ - b χ)])) /
   (n (b (2 χ - 2 ⅇ^χ χ + 2 χ^2 - b χ^2 + b ⅇ^χ χ^2 - 2 b (-1 + ⅇ^χ) χ Log[1 - (b (-1 + ⅇ^χ)) / ((-1 + b) χ)] -
          2 (-1 + b) (-1 + ⅇ^χ) χ (Log[b] + Log[-1 + ⅇ^χ] - Log[b (-1 + ⅇ^χ - χ) + χ]) +
          2 (-1 + ⅇ^χ - χ) Log[(ⅇ^χ (b (-1 + ⅇ^χ - χ) + χ)) / (-b + ⅇ^χ (b + χ - b χ))] -
          2 b (-1 + ⅇ^χ) PolyLog[2, (b (-1 + ⅇ^χ)) / ((-1 + b) χ)] -
          2 (-1 + b) (-1 + ⅇ^χ) PolyLog[2, ((-1 + b) χ) / (b (-1 + ⅇ^χ))] +
          2 (-1 + b) (-1 + ⅇ^χ) PolyLog[2, ((-1 + b) ⅇ^χ χ) / (b (-1 + ⅇ^χ))] +
          2 b (-1 + ⅇ^χ) PolyLog[2, -((b - b ⅇ^-χ) / (χ - b χ))])^2 +
       4 (χ - b χ + Log[b (-1 + ⅇ^χ - χ) + χ] - Log[-b + ⅇ^χ (b + χ - b χ)])
        (-χ + 2 b χ + 2 ⅇ^χ χ - 4 b ⅇ^χ χ - ⅇ^(2 χ) χ + 2 b ⅇ^(2 χ) χ + 2 b χ^2 - 2 b ⅇ^χ χ^2 + b χ^3 + ⅇ^χ χ^3 - b ⅇ^χ χ^3 -
         2 b (-1 + ⅇ^χ) (-1 + ⅇ^χ - χ) χ (Log[b] + Log[-1 + ⅇ^χ] - Log[b (-1 + ⅇ^χ - χ) + χ]) +
         b (-1 + ⅇ^χ)^2 χ^2 (Log[b (-1 + ⅇ^χ)] - Log[b (-1 + ⅇ^χ - χ) + χ]) -
         b (1 - ⅇ^χ + χ)^2 Log[(ⅇ^χ (b (-1 + ⅇ^χ - χ) + χ)) / (-b + ⅇ^χ (b + χ - b χ))] +
         2 b (-1 + ⅇ^χ) (1 + ⅇ^χ (-1 + χ)) PolyLog[2, ((-1 + b) χ) / (b (-1 + ⅇ^χ))] +
         2 b (-1 + ⅇ^χ) (-1 + ⅇ^χ - χ) PolyLog[2, ((-1 + b) ⅇ^χ χ) / (b (-1 + ⅇ^χ))] +
         2 b (-1 + ⅇ^χ)^2 PolyLog[3, ((-1 + b) χ) / (b (-1 + ⅇ^χ))] -
         2 b (-1 + ⅇ^χ)^2 PolyLog[3, ((-1 + b) ⅇ^χ χ) / (b (-1 + ⅇ^χ))]))))
```

Limit for zero background:

```
In[13]:= Assuming[pAssumptions, FullSimplify[1 / Limit[1 / στSQ, b → 0, Direction → "FromAbove"]]]
(* The limits of 1/στSQ are somehow much faster to compute. *)
```

... Limit: Warning: Assumptions that involve the limit variable are ignored.

```
Out[13]= (4 τ^2 (2 + χ^2 - 2 Cosh[χ]) Sinh[χ / 2]) /
  (n (-4 χ^3 Cosh[χ / 2] + (12 + 12 χ^2 + χ^4) Sinh[χ / 2] - 4 Sinh[(3 χ) / 2]))
```

Limit for infinite pulse period:

```
In[14]:= 1 / Limit[1 / στSQ, χ → ∞, Assumptions → pAssumptions, Direction → "FromBelow"]
```

... Limit: Warning: Assumptions that involve the limit variable are ignored.

Out[14]= $\frac{\tau^2}{n - b\,n}$

Independence of CRLB of τ and b on N

To demonstrate that the CRLB of τ and b are independent of the uncertainty of the number of photons N, we evaluate the full ($\theta = \{\tau, b, N\}$) Fisher matrix $f = \begin{pmatrix} f_{\tau\tau} & f_{\tau b} & f_{\tau N} \\ f_{\tau b} & f_{bb} & f_{bN} \\ f_{\tau N} & f_{bN} & f_{NN} \end{pmatrix}$ and show that the off-diagonal entries with N, i.e. $f_{\tau N}$ and f_{bN} are zero.

```
In[15]:= fτN =
  Integrate[(∂τ Log[n * p]) * (∂n Log[n * p]) * (n * p), {t, 0, T}, Assumptions → pAssumptions]
Out[15]= 0

In[16]:= fbN =
  Integrate[(∂b Log[n * p]) * (∂n Log[n * p]) * (n * p), {t, 0, T}, Assumptions → pAssumptions]
Out[16]= 0
```

For a infinite pulse period, all diagonal entries vanish:

```
In[17]:= Limit[fτb, T → ∞, Assumptions → pAssumptions, Direction → "FromBelow"]
```

... Limit: Warning: Assumptions that involve the limit variable are ignored.

```
Out[17]= 0
```

CRLB of the background estimation

In[18]:=
```
σbSQ = SimplifyCRLB[Last@Last@Inverse[f //. {T → χ τ}]]
```

Out[18]=

$$
\begin{aligned}
\Big(&4\,(-1+b)^2\,\chi\,\Big(-\chi+2\,b\,\chi+2\,e^{\chi}\,\chi-4\,b\,e^{\chi}\,\chi-e^{2\chi}\,\chi+2\,b\,e^{2\chi}\,\chi+2\,b\,\chi^2-2\,b\,e^{\chi}\,\chi^2+b\,\chi^3+e^{\chi}\,\chi^3-\\
&b\,e^{\chi}\,\chi^3-2\,b\,(-1+e^{\chi})\,(-1+e^{\chi}-\chi)\,\chi\,\big(\mathrm{Log}[b]+\mathrm{Log}[-1+e^{\chi}]-\mathrm{Log}[b\,(-1+e^{\chi}-\chi)+\chi]\big)+\\
&b\,(-1+e^{\chi})^2\,\chi^2\,\big(\mathrm{Log}[b\,(-1+e^{\chi})]-\mathrm{Log}[b\,(-1+e^{\chi}-\chi)+\chi]\big)-\\
&b\,(1-e^{\chi}+\chi)^2\,\mathrm{Log}\Big[\frac{e^{\chi}\,(b\,(-1+e^{\chi}-\chi)+\chi)}{-b+e^{\chi}\,(b+\chi-b\,\chi)}\Big]+2\,b\,(-1+e^{\chi})\,(1+e^{\chi}\,(-1+\chi))\\
&\mathrm{PolyLog}\Big[2,\frac{(-1+b)\,\chi}{b\,(-1+e^{\chi})}\Big]+2\,b\,(-1+e^{\chi})\,(-1+e^{\chi}-\chi)\,\mathrm{PolyLog}\Big[2,\frac{(-1+b)\,e^{\chi}\,\chi}{b\,(-1+e^{\chi})}\Big]+\\
&2\,b\,(-1+e^{\chi})^2\,\mathrm{PolyLog}\Big[3,\frac{(-1+b)\,\chi}{b\,(-1+e^{\chi})}\Big]-2\,b\,(-1+e^{\chi})^2\,\mathrm{PolyLog}\Big[3,\frac{(-1+b)\,e^{\chi}\,\chi}{b\,(-1+e^{\chi})}\Big]\Big)\Big)\Big/\\
\Big(&n\,\Big(\Big(2\,\chi-2\,e^{\chi}\,\chi+2\,\chi^2-b\,\chi^2+b\,e^{\chi}\,\chi^2-2\,b\,(-1+e^{\chi})\,\chi\,\mathrm{Log}\Big[1-\frac{b\,(-1+e^{\chi})}{(-1+b)\,\chi}\Big]-\\
&2\,(-1+b)\,(-1+e^{\chi})\,\chi\,\big(\mathrm{Log}[b]+\mathrm{Log}[-1+e^{\chi}]-\mathrm{Log}[b\,(-1+e^{\chi}-\chi)+\chi]\big)+\\
&2\,(-1+e^{\chi}-\chi)\,\mathrm{Log}\Big[\frac{e^{\chi}\,(b\,(-1+e^{\chi}-\chi)+\chi)}{-b+e^{\chi}\,(b+\chi-b\,\chi)}\Big]-2\,b\,(-1+e^{\chi})\,\mathrm{PolyLog}\Big[2,\frac{b\,(-1+e^{\chi})}{(-1+b)\,\chi}\Big]-\\
&2\,(-1+b)\,(-1+e^{\chi})\,\mathrm{PolyLog}\Big[2,\frac{(-1+b)\,\chi}{b\,(-1+e^{\chi})}\Big]+2\,(-1+b)\,(-1+e^{\chi})\\
&\mathrm{PolyLog}\Big[2,\frac{(-1+b)\,e^{\chi}\,\chi}{b\,(-1+e^{\chi})}\Big]+2\,b\,(-1+e^{\chi})\,\mathrm{PolyLog}\Big[2,-\frac{b-b\,e^{-\chi}}{\chi-b\,\chi}\Big]\Big)^2+\\
&\frac{1}{b}4\,\big((-1+b)\,\chi-\mathrm{Log}[b\,(-1+e^{\chi}-\chi)+\chi]+\mathrm{Log}[-b+e^{\chi}\,(b+\chi-b\,\chi)]\big)\\
&\Big(\chi-2\,b\,\chi-2\,e^{\chi}\,\chi+4\,b\,e^{\chi}\,\chi+e^{2\chi}\,\chi-2\,b\,e^{2\chi}\,\chi-2\,b\,\chi^2+2\,b\,e^{\chi}\,\chi^2-b\,\chi^3-e^{\chi}\,\chi^3+b\,e^{\chi}\,\chi^3+\\
&2\,b\,(-1+e^{\chi})\,(-1+e^{\chi}-\chi)\,\chi\,\big(\mathrm{Log}[b]+\mathrm{Log}[-1+e^{\chi}]-\mathrm{Log}[b\,(-1+e^{\chi}-\chi)+\chi]\big)-\\
&b\,(-1+e^{\chi})^2\,\chi^2\,\big(\mathrm{Log}[b\,(-1+e^{\chi})]-\mathrm{Log}[b\,(-1+e^{\chi}-\chi)+\chi]\big)+\\
&b\,(1-e^{\chi}+\chi)^2\,\mathrm{Log}\Big[\frac{e^{\chi}\,(b\,(-1+e^{\chi}-\chi)+\chi)}{-b+e^{\chi}\,(b+\chi-b\,\chi)}\Big]-2\,b\,(-1+e^{\chi})\,(1+e^{\chi}\,(-1+\chi))\\
&\mathrm{PolyLog}\Big[2,\frac{(-1+b)\,\chi}{b\,(-1+e^{\chi})}\Big]-2\,b\,(-1+e^{\chi})\,(-1+e^{\chi}-\chi)\,\mathrm{PolyLog}\Big[2,\frac{(-1+b)\,e^{\chi}\,\chi}{b\,(-1+e^{\chi})}\Big]-\\
&2\,b\,(-1+e^{\chi})^2\,\mathrm{PolyLog}\Big[3,\frac{(-1+b)\,\chi}{b\,(-1+e^{\chi})}\Big]+2\,b\,(-1+e^{\chi})^2\,\mathrm{PolyLog}\Big[3,\frac{(-1+b)\,e^{\chi}\,\chi}{b\,(-1+e^{\chi})}\Big]\Big)\Big)\Big)
\end{aligned}
$$

Limit for zero background:

In[19]:=
```
Assuming[pAssumptions, FullSimplify[1 / Limit[1 / σbSQ, b → 0, Direction → "FromAbove"]]]
```

... Limit: Warning: Assumptions that involve the limit variable are ignored.

Out[19]=

$$
-\frac{2\,\chi^2\,(2+\chi^2-2\,\mathrm{Cosh}[\chi])}{n\,(12+12\,\chi^2+\chi^4-(16+12\,\chi^2+\chi^4)\,\mathrm{Cosh}[\chi]+4\,\mathrm{Cosh}[2\,\chi]+4\,\chi^3\,\mathrm{Sinh}[\chi])}
$$

Limit for infinite pulse period:

In[20]:=
```
Limit[σbSQ, {χ → ∞}, Assumptions → pAssumptions]
```

Limit: Warning: Assumptions that involve the limit variable are ignored.

Out[20]= $-\frac{(-1+b)\,b}{n}$
