# Supplementary material for: Advanced Data Analysis for Fluorescence-Lifetime Single-Molecule Localization Microscopy
Source: Front Bioinform. 2021 Nov 19;1:740281. doi: 10.3389/fbinf.2021.740281 (PMC9581058; doi:10.3389/fbinf.2021.740281)
Supplement: Supplementary file 2 [file DataSheet1.PDF]

frontiers

Supplementary Material

1 SUPPLEMENTARY EQUATIONS

1.1 Cramér-Rao lower bound with unknown background

Cramér-Rao lower bound when lifetime τ and background fraction b need to estimated simultaneously:

$$
\begin{aligned}
\sigma_\tau^2 =& \frac{\tau^2}{N} 4\left(e^\chi-1\right)^2 \chi\left((b-1)\chi-\log\left(b\left(-\chi+e^\chi-1\right)+\chi\right)+\log\left(b\left(-e^\chi\chi+e^\chi-1\right)+e^\chi\chi\right)\right) \\
&\Bigg(-b\bigg(2b\left(e^\chi-1\right)\mathrm{Li}_2\left(\frac{b\left(-1+e^\chi\right)}{(b-1)\chi}\right)+2(b-1)\left(e^\chi-1\right)\mathrm{Li}_2\left(\frac{(b-1)\chi}{b\left(-1+e^\chi\right)}\right) \\
&-2(b-1)\left(e^\chi-1\right)\mathrm{Li}_2\left(\frac{(b-1)e^\chi\chi}{b\left(-1+e^\chi\right)}\right)-2b\left(e^\chi-1\right)\mathrm{Li}_2\left(-\frac{b-be^{-\chi}}{\chi-b\chi}\right) \\
&-be^\chi\chi^2+b\chi^2+2(b-1)\left(e^\chi-1\right)\chi\log\left(b\left(e^\chi-1\right)\right)-2b\left(e^\chi-1\right)\chi\log(\chi-b\chi) \\
&+\left(2\left(e^\chi-1\right)\chi+2\right)\log\left(b\left(-\chi+e^\chi-1\right)+\chi\right)-\log\left(\left(be^\chi(\chi-1)+b-e^\chi\chi\right)^2\right) \\
&-2\left(e^\chi-\chi\right)\left(-\log\left(be^\chi(\chi-1)+b-e^\chi\chi\right)+\log\left(b\left(\chi-e^\chi+1\right)-\chi\right)+\chi\right)-2\chi^2+2e^\chi\chi\bigg)^2 \\
&+4\left((b-1)\chi-\log\left(b\left(-\chi+e^\chi-1\right)+\chi\right)+\log\left(b\left(-e^\chi\chi+e^\chi-1\right)+e^\chi\chi\right)\right) \\
&\bigg(2b\left(e^\chi-1\right)\left(e^\chi(\chi-1)+1\right)\mathrm{Li}_2\left(\frac{(b-1)\chi}{b\left(-1+e^\chi\right)}\right)+2b\left(e^\chi-1\right)\left(-\chi+e^\chi-1\right)\mathrm{Li}_2\left(\frac{(b-1)e^\chi\chi}{b\left(-1+e^\chi\right)}\right) \\
&+2b\left(e^\chi-1\right)^2\mathrm{Li}_3\left(\frac{(b-1)\chi}{b\left(-1+e^\chi\right)}\right)-2b\left(e^\chi-1\right)^2\mathrm{Li}_3\left(\frac{(b-1)e^\chi\chi}{b\left(-1+e^\chi\right)}\right) \\
&-be^\chi\chi^3+b\chi^3-2be^\chi\chi^2+2b\chi^2+b\left(e^\chi-1\right)^2\chi^2\left(\log\left(b\left(e^\chi-1\right)\right)-\log\left(b\left(-\chi+e^\chi-1\right)+\chi\right)\right) \\
&-4be^\chi\chi+2be^{2\chi}\chi+2b\chi-2b\left(e^\chi-1\right)\left(-\chi+e^\chi-1\right)\chi \\
&\left(-\log\left(b\left(-\chi+e^\chi-1\right)+\chi\right)+\log(b)+\log\left(e^\chi-1\right)\right) \\
&-b\left(\chi-e^\chi+1\right)^2\log\left(\frac{e^\chi\left(b\left(-\chi+e^\chi-1\right)+\chi\right)}{e^\chi(b(-\chi)+b+\chi)-b}\right)+e^\chi\chi^3+2e^\chi\chi-e^{2\chi}\chi-\chi\bigg)\Bigg)^{-1}
\end{aligned}
\tag{S1}
$$

with $\chi = T/\tau$. Li_n denotes the nth polylogarithm.

Limit for no background ($b \to 0$):

$$
\lim_{b\to 0}\sigma_\tau^2 = \frac{\tau^2}{N}\frac{4\sinh\left(\frac{\chi}{2}\right)\left(\chi^2-2\cosh(\chi)+2\right)}{\left(\chi^4+12\chi^2+12\right)\sinh\left(\frac{\chi}{2}\right)-4\chi^3\cosh\left(\frac{\chi}{2}\right)-4\sinh\left(\frac{3\chi}{2}\right)}
\tag{S2}
$$

Limit for an infinite measurement window ($T \to \infty$):

$$\lim_{\chi \to \infty} \sigma_\tau^2 = \frac{\tau^2}{N} \frac{1}{1-b} \tag{S3}$$

1.2 Cramér-Rao lower bound with known background

Cramér-Rao lower bound when the background fraction b is known and only the lifetime τ needs to estimated:

$$\begin{aligned}
\sigma_\tau^2 =& \frac{\tau^2}{N} \left(e^\chi - 1\right)^2 (-\chi) \\
& \left(2b\left(e^\chi - 1\right)\left(e^\chi(\chi - 1) + 1\right) \mathrm{Li}_2\left(\frac{(b-1)\chi}{b\left(-1+e^\chi\right)}\right) + 2b\left(e^\chi - 1\right)\left(-\chi + e^\chi - 1\right) \mathrm{Li}_2\left(\frac{(b-1)e^\chi\chi}{b\left(-1+e^\chi\right)}\right) \right. \\
& + 2b\left(e^\chi - 1\right)^2 \mathrm{Li}_3\left(\frac{(b-1)\chi}{b\left(-1+e^\chi\right)}\right) - 2b\left(e^\chi - 1\right)^2 \mathrm{Li}_3\left(\frac{(b-1)e^\chi\chi}{b\left(-1+e^\chi\right)}\right) - be^\chi\chi^3 + b\chi^3 - 2be^\chi\chi^2 \\
& + 2b\chi^2 + b\left(e^\chi - 1\right)^2 \chi^2 \left(\log\left(b\left(e^\chi - 1\right)\right) - \log\left(b\left(-\chi + e^\chi - 1\right) + \chi\right)\right) \\
& - 4be^\chi\chi + 2be^{2\chi}\chi + 2b\chi - 2b\left(e^\chi - 1\right)\left(-\chi + e^\chi - 1\right)\chi \\
& \left(-\log\left(b\left(-\chi + e^\chi - 1\right) + \chi\right) + \log(b) + \log\left(e^\chi - 1\right)\right) \\
& \left. - b\left(\chi - e^\chi + 1\right)^2 \log\left(\frac{e^\chi\left(b\left(-\chi + e^\chi - 1\right) + \chi\right)}{e^\chi(b(-\chi) + b + \chi) - b}\right) + e^\chi\chi^3 + 2e^\chi\chi - e^{2\chi}\chi - \chi \right)^{-1}
\end{aligned} \tag{S4}$$

Limit for no background ($b \to 0$):

$$\lim_{b \to 0} \sigma_\tau^2 = \frac{\tau^2}{N} \frac{2(1 - \cosh(\chi))}{2 + \chi^2 - 2\cosh(\chi)} \tag{S5}$$

Limit for an infinite measurement window ($T \to \infty$):

$$\lim_{\chi \to \infty} \sigma_\tau^2 = \frac{\tau^2}{N} \frac{1}{1-b} \tag{S6}$$

2 SUPPLEMENTARY TABLES AND FIGURES

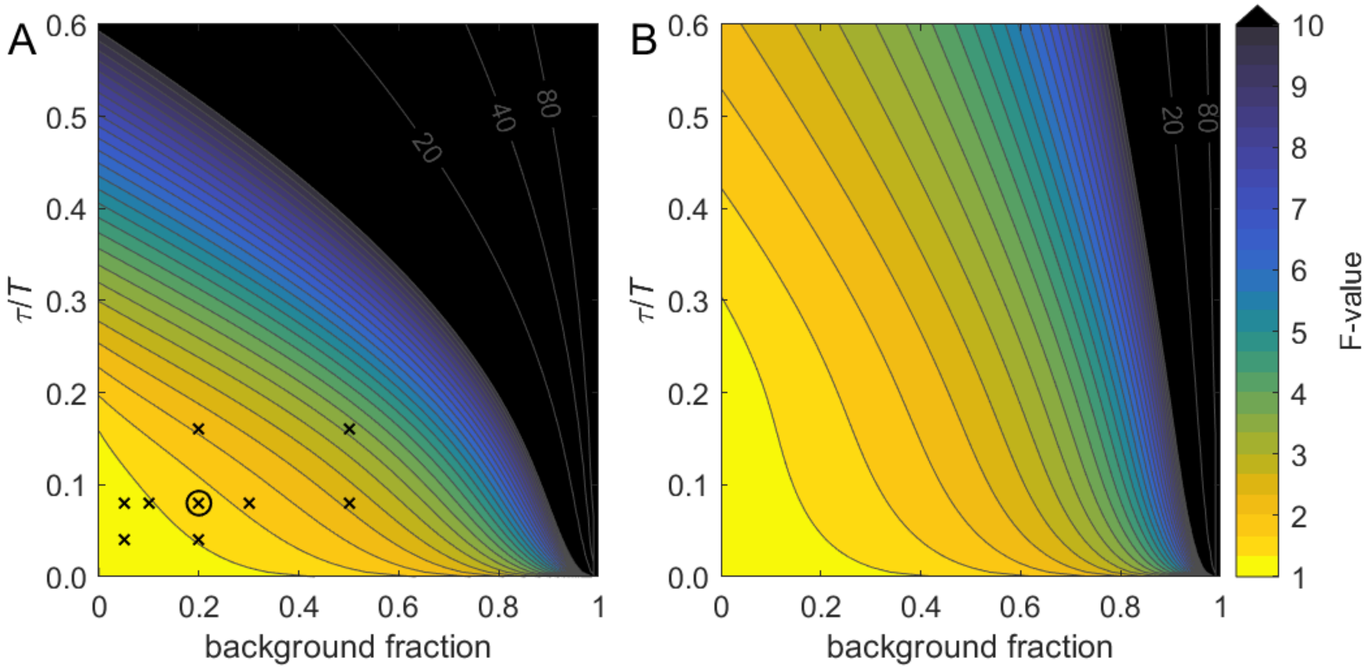

Figure S1. Effect of background and finite measurement period on the lifetime uncertainty: The F-value, defined as the ratio of the CRLB σ_τ to the shot-noise limit ($\lim_{T\to\infty} \lim_{b\to 0} \sigma_\tau = \tau/\sqrt{N}$), only depends on the ratio τ/T but not τ and T separately. For **(A)**, it is assumed the background needs to be estimated, for **(B)** that the background is known. A known background decreases the F-value and therefore reduces the theoretical lifetime uncertainty. The $\circ$ marker in (A) indicates the parameters of the simulation presented in figure 1, the $\times$ markers the parameters for the additional simulations in the supplementary figures.

τ (ns)	b	T (ns)	ΔT (ns)	Figure
2.0	0.20	25.0	0.016	S3
2.0	0.20	25.0	**0.160**	S4
2.0	0.20	**12.5**	0.016	S5
**1.0**	0.20	25.0	0.016	S6
2.0	**0.10**	25.0	0.016	S7
2.0	**0.05**	25.0	0.016	S8
2.0	**0.30**	25.0	0.016	S9
2.0	**0.50**	25.0	0.016	S10
2.0	**0.50**	**12.5**	0.016	S11
**1.0**	**0.05**	25.0	0.016	S12

Table S1. List of additional simulations with an overview of their parameters. Parameter different to the conditions in the first row are highlighted in bold.

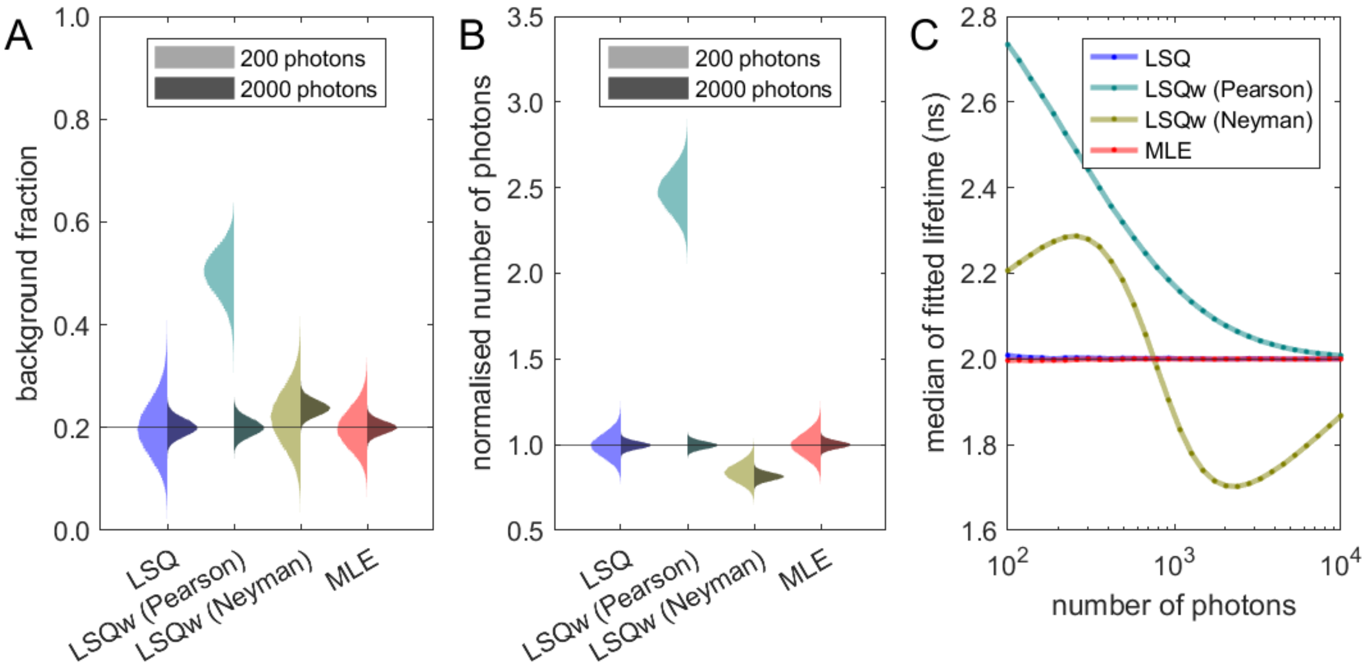

Figure S2. Distribution of the estimated background **(A)** and number of photons **(B)** for the simulated decay curves corresponding to figure 1. For the MLE, the number of photons is not a fit parameter but estimated by the number of photons in the simulated decay. **(C)** Median estimated lifetime in dependency of the number of photons in the decay for the different estimators.

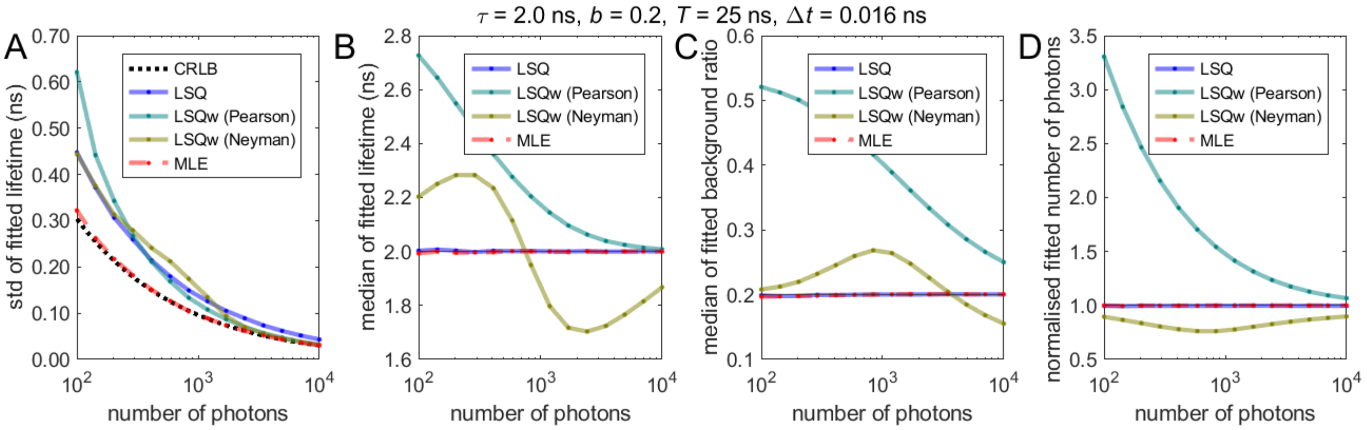

Figure S3. Performance of different lifetime estimators in dependence of the number of photons: Based on simulated decays with lifetime $\tau = 2$ ns, background $b = 20\,\%$, repetition period $T = 25$ ns, and time resolution $\Delta t = 0.016$ ns. These parameters are equivalent to the simulation in figure 1. For each photon number, 10^4 decays were simulated and fitted. **(A)** Standard deviation of the fitted lifetimes and analytical CRLB. **(B)** Median of the fitted lifetimes. **(C)** Median of the fitted background. **(D)** Median of the fitted number of photons normalized by the expected number of photons.

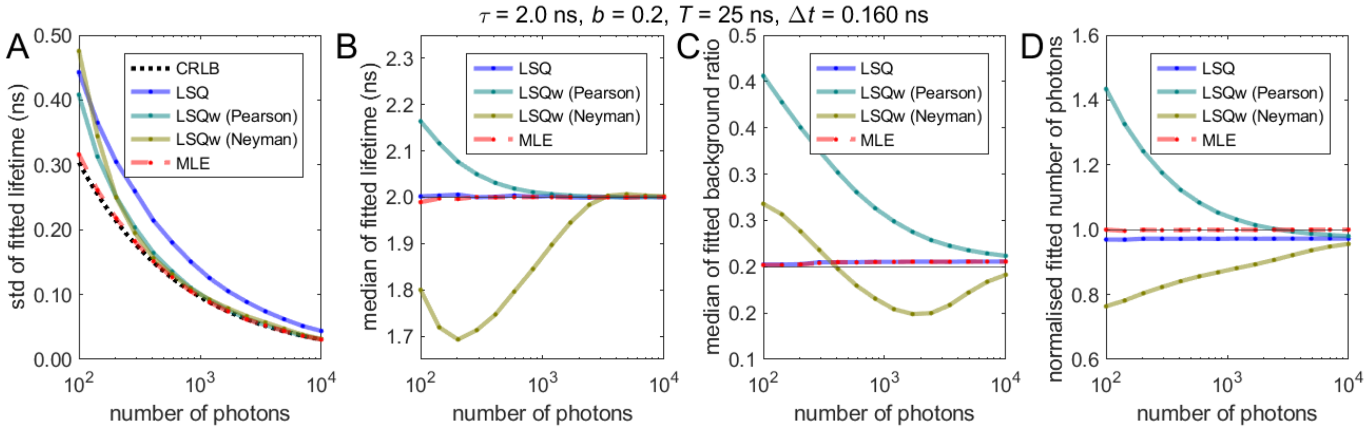

Figure S4. Same as figure S3, but for a lower time resolution. Simulation parameter: lifetime $\tau = 2$ ns, background $b = 20\,\%$, repetition period $T = 25$ ns, and time resolution $\Delta t = 0.160$ ns.

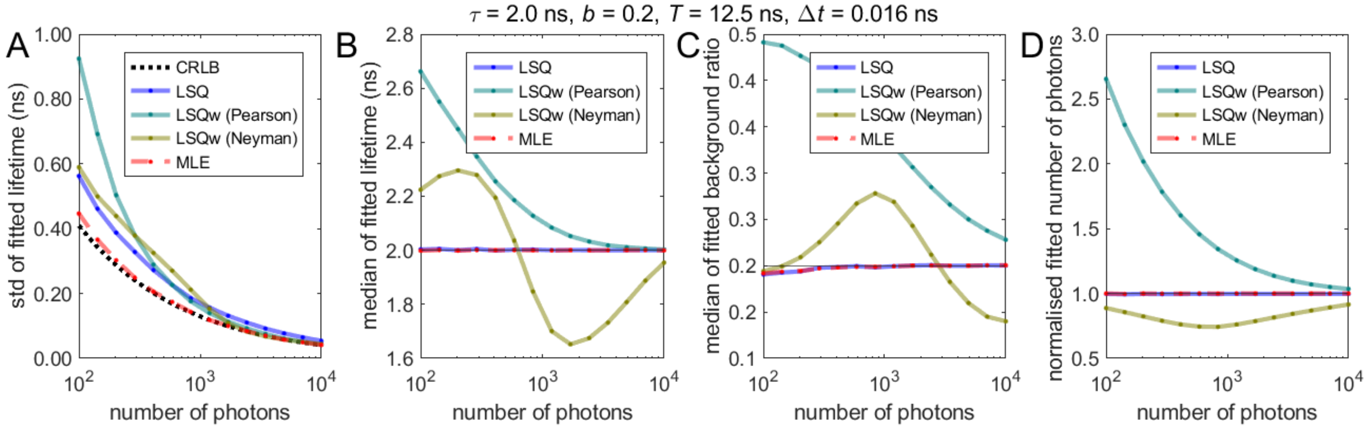

Figure S5. Same as figure S3, but for a decreased repetition period. Simulation parameter: lifetime $\tau = 2$ ns, background $b = 20\,\%$, repetition period $T = 12.5$ ns, and time resolution $\Delta t = 0.016$ ns.

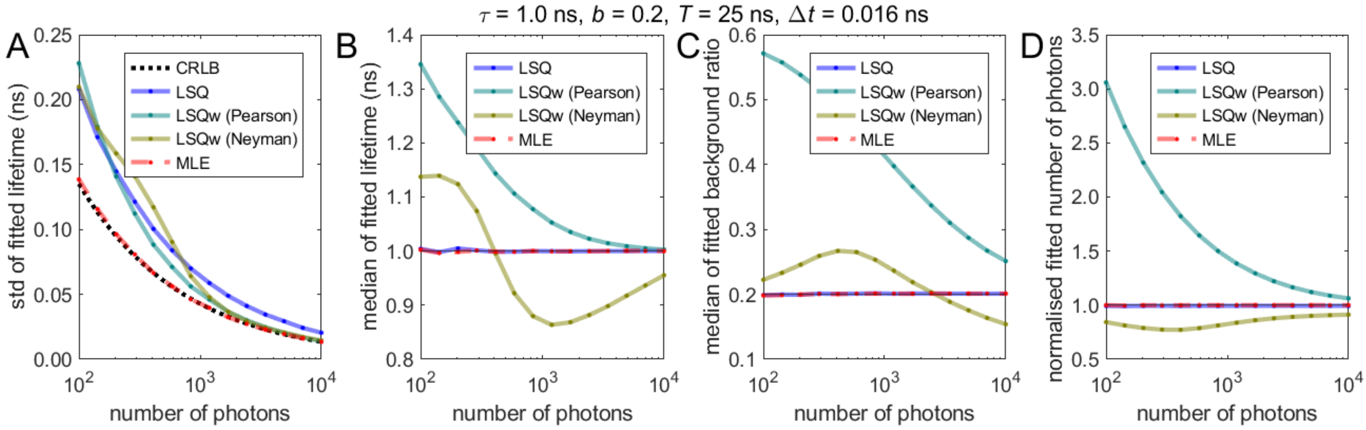

Figure S6. Same as figure S3, but for a decreased lifetime. Simulation parameter: lifetime $\tau = 1$ ns, background $b = 20\,\%$, repetition period $T = 25$ ns, and time resolution $\Delta t = 0.016$ ns.

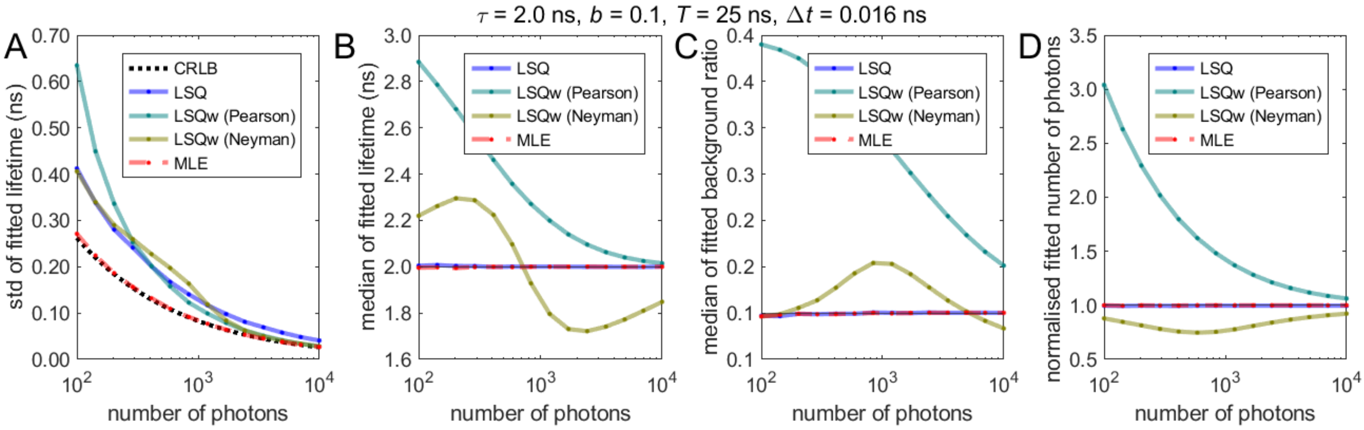

Figure S7. Same as figure S3, but for a lower background. Simulation parameter: lifetime $\tau = 2$ ns, background $b = 10\,\%$, repetition period $T = 25$ ns, and time resolution $\Delta t = 0.016$ ns.

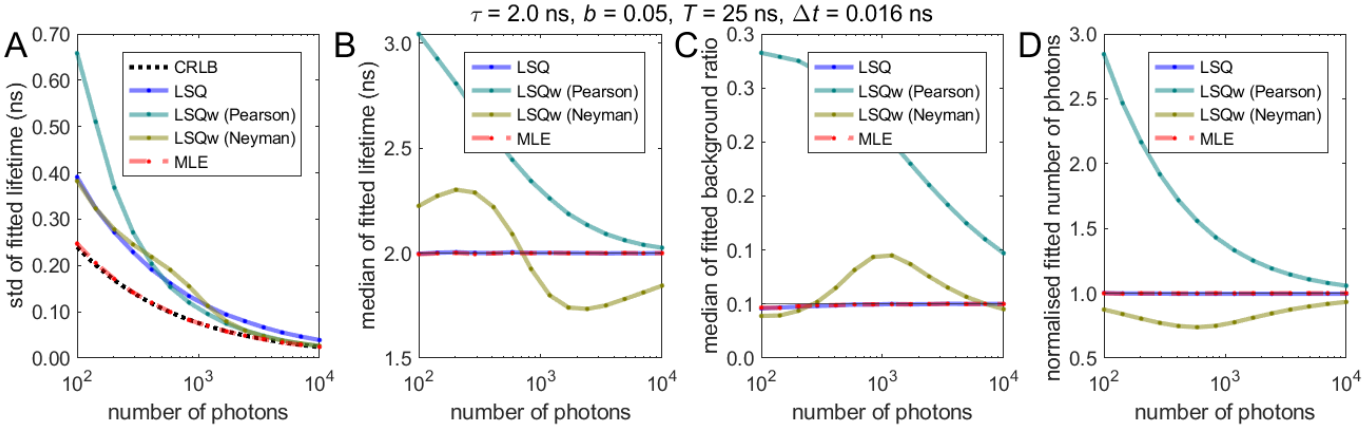

Figure S8. Same as figure S3, but for a lower background. Simulation parameter: lifetime $\tau = 2$ ns, background $b = 5\,\%$, repetition period $T = 25$ ns, and time resolution $\Delta t = 0.016$ ns.

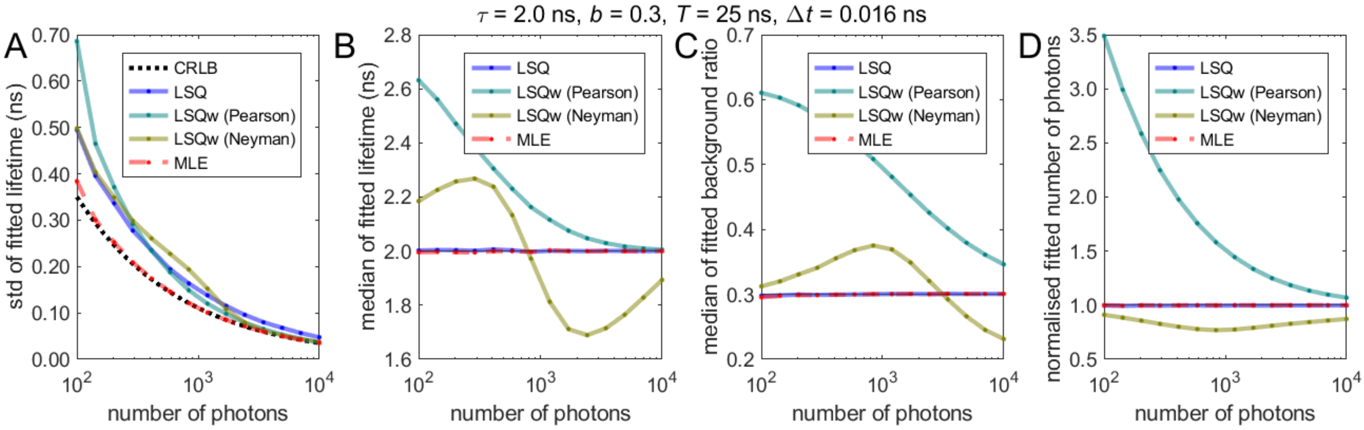

Figure S9. Same as figure S3, but for a higher background. Simulation parameter: lifetime $\tau = 2$ ns, background $b = 30\,\%$, repetition period $T = 25$ ns, and time resolution $\Delta t = 0.016$ ns.

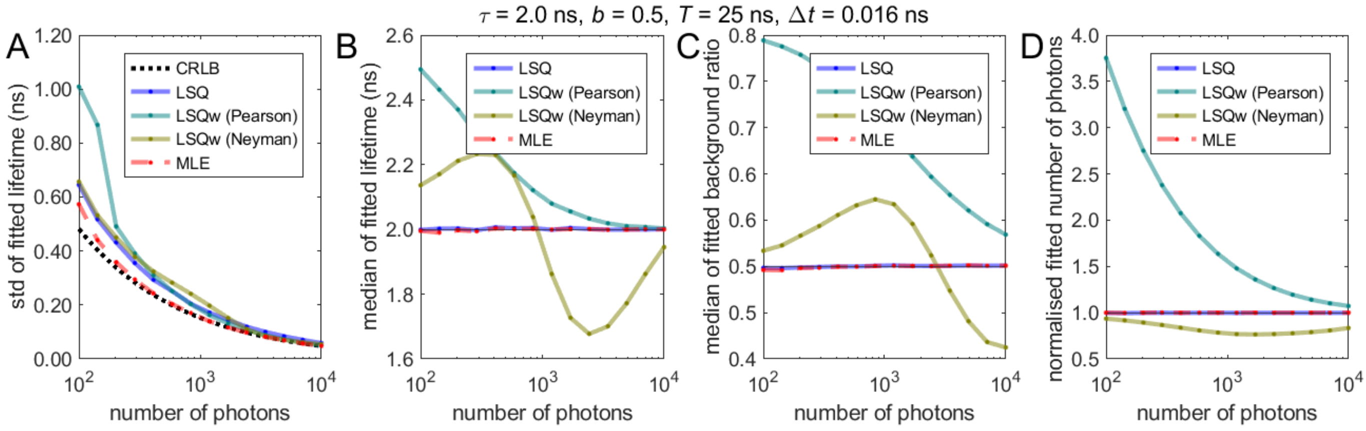

Figure S10. Same as figure S3, but for a higher background. Simulation parameter: lifetime $\tau = 2$ ns, background $b = 50\,\%$, repetition period $T = 25$ ns, and time resolution $\Delta t = 0.016$ ns.

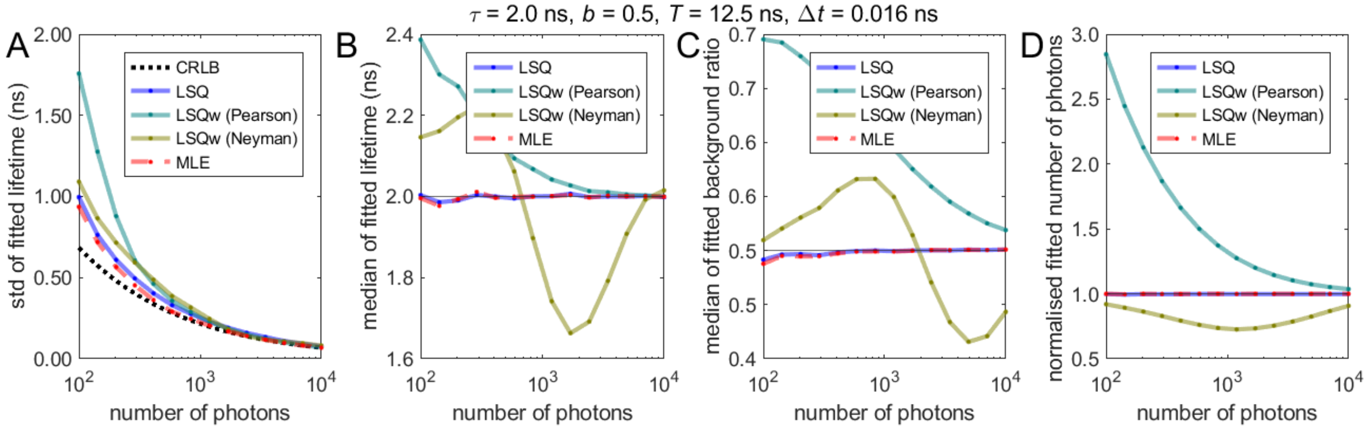

Figure S11. Same as figure S3, but for a higher background and decreased repetition period. Simulation parameter: lifetime $\tau = 2$ ns, background $b = 50\,\%$, repetition period $T = 12.5$ ns, and time resolution $\Delta t = 0.016$ ns.

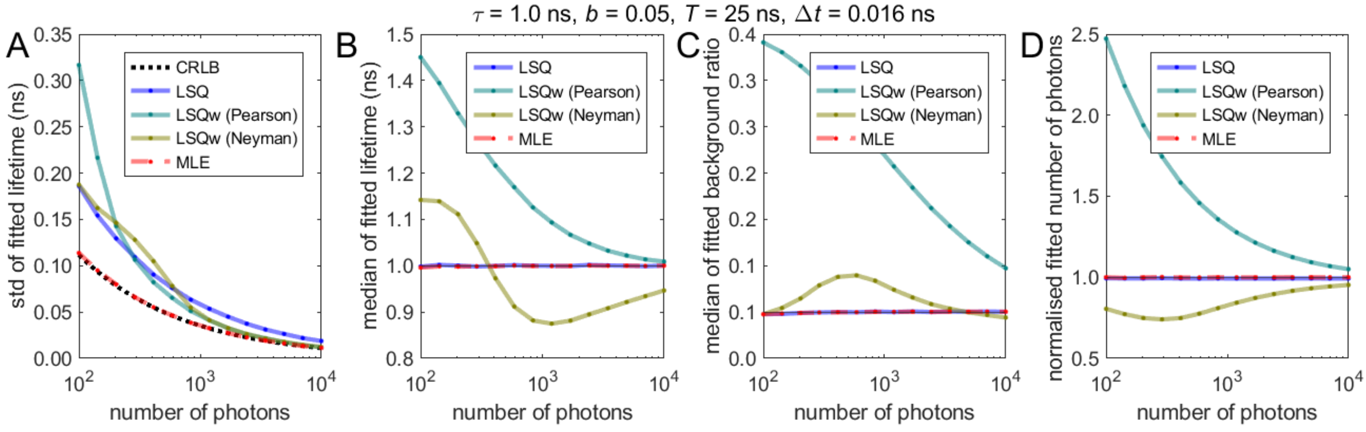

Figure S12. Same as figure S3, but for a lower background and lower lifetime. Simulation parameter: lifetime $\tau = 1$ ns, background $b = 5\,\%$, repetition period $T = 25$ ns, and time resolution $\Delta t = 0.016$ ns.

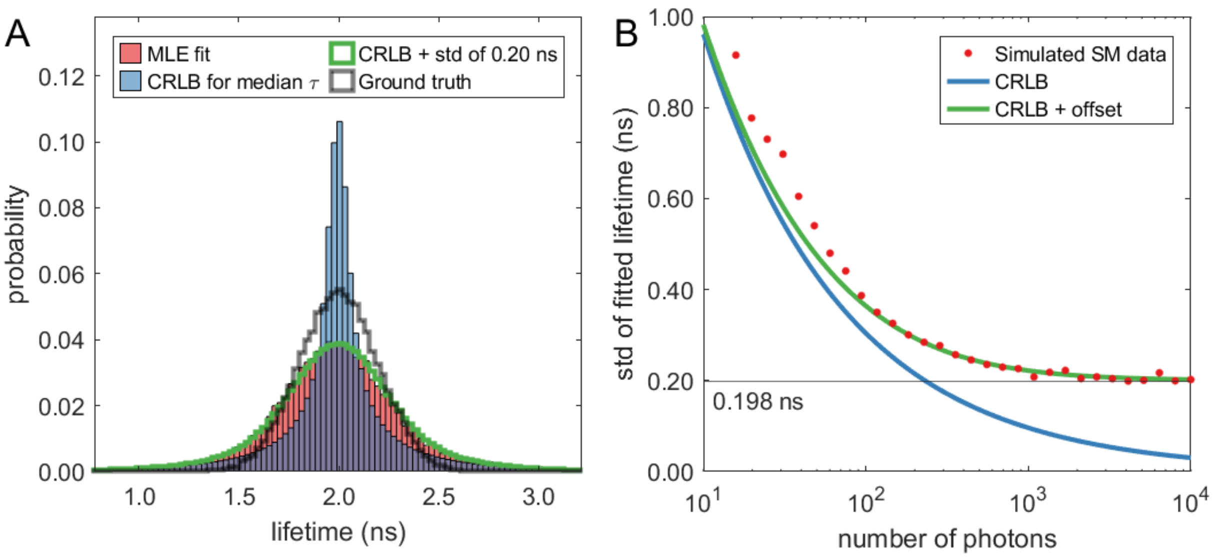

Figure S13. Lifetime distribution for simulated lifetime variations in the sample: Assuming normal distributed single-molecule lifetimes with mean 2.0 ns and standard deviation $\sigma_{\text{sample}} = 0.20$ ns, the TCSPC-histograms of 1000 molecules were simulated ($b = 0.20$, $T = 25$ ns, and $\Delta t = 0.016$ ns) and fitted with an MLE grid search. This was repeated for 30 different average photon numbers. **(A)** Distribution of the fitted lifetime values (red), expected CRLB-limited distribution (blue) for lifetime 2 ns and the simulated photon numbers, CRLB-limited distribution with an additional broadening (green), and lifetime values of the simulated molecules (gray). **(B)** Dependence of the standard deviation of the fitted lifetimes on the number of photons. With an increasing number of photons, the standard deviation (red) approaches the simulated variance of the sample $\sigma_{\text{sample}} = 0.2$ ns. Based on the simulated data, an intrinsic variance of 0.198 ns is estimated which agrees well with the simulated variance. The green line represents $(\sigma_\tau^2 + \sigma_{\text{sample}}^2)^{1/2}$ with variance σ_τ^2 calculated by the CRLB (blue) and the intrinsic variance of the sample σ_{sample}^2.

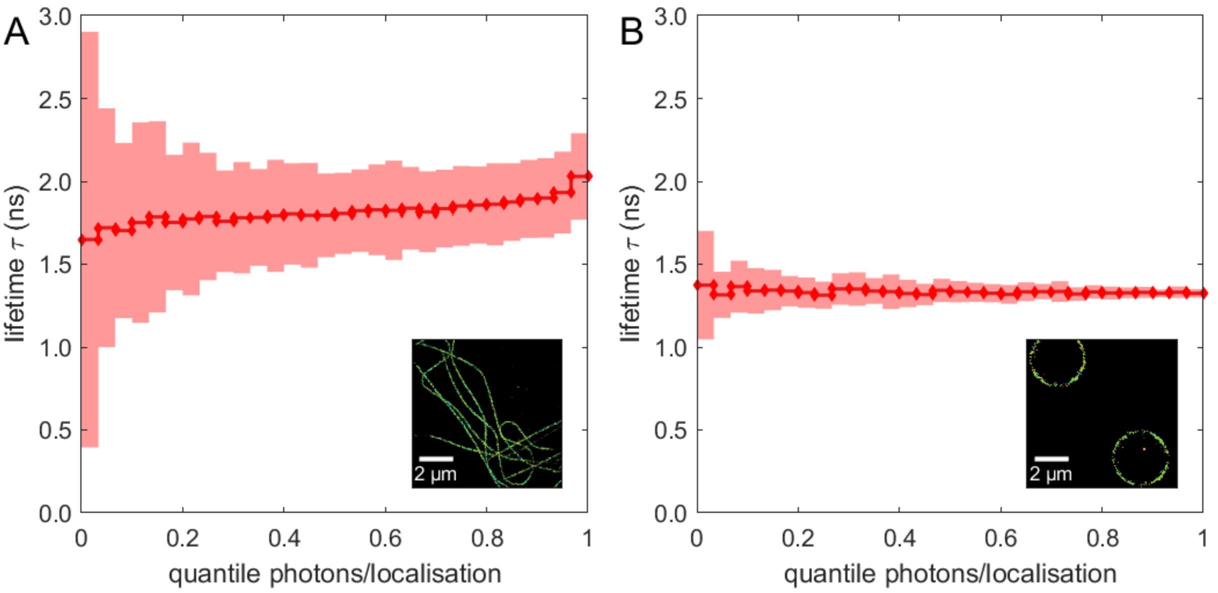

Figure S14. Number of photon dependence of the single-molecule lifetime distribution for the two data sets presented in figure 3: The data of the single molecules was ordered by number of photons in the TCSPC histogram and divided into 30 group. For each group, the mean and standard deviation were calculated. As indicated by the insets, **(A)** is based on a dSTROM measurement of a cell, **(B)** on a measurement of DNA-functionalized micro-beads.

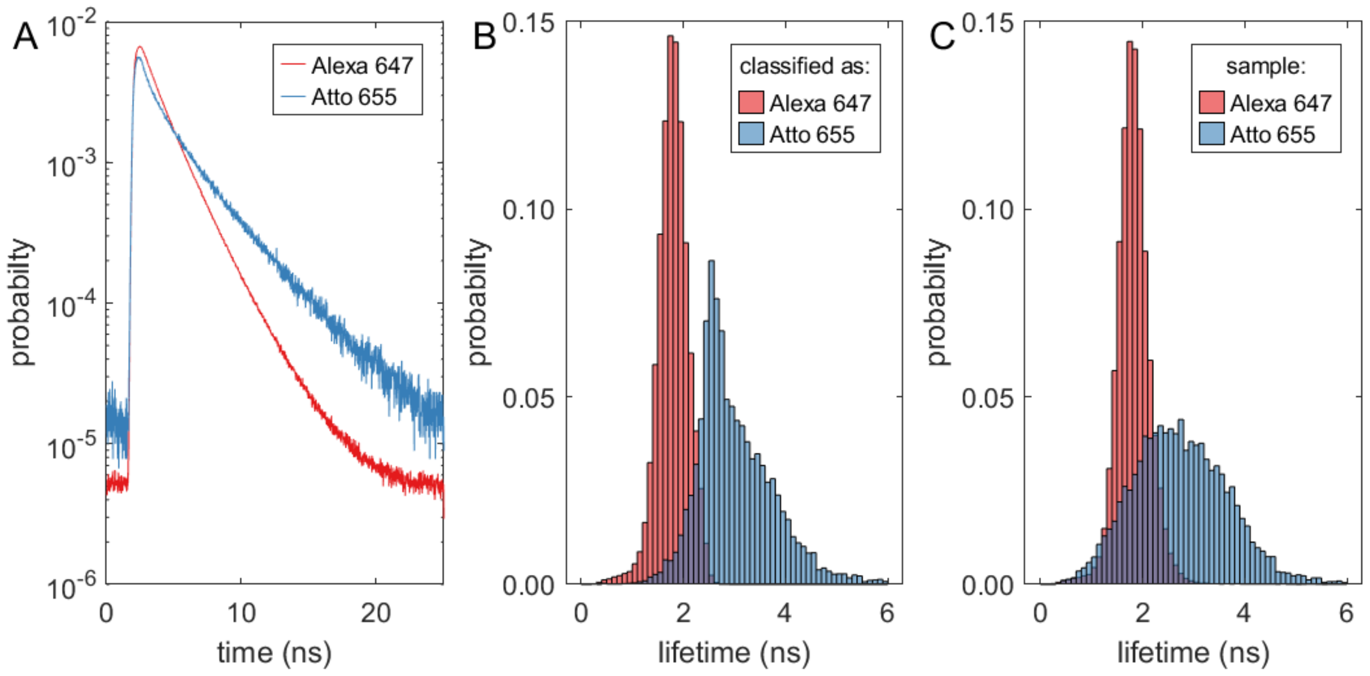

Figure S15. Classification by pattern matching: **(A)** normalized reference decay curves from samples with only Alexa 647 or Atto 655. **(B)** lifetime distribution of the single molecules classified as either Alexa 647 or Atto 655. Data from multiple samples with Alexa 647 or Atto 655 was combined and classified based on the reference curves in (A). **(C)** lifetime distribution of the single molecules from samples with only Alexa 647 or Atto 655.
